# The Tides of Enceladus' Porous Core

**DOI:** 10.1029/2021JE007117

**Published:** 2022-05-24

**Authors:** Marc Rovira‐Navarro, Richard F. Katz, Yang Liao, Wouter van der Wal, Francis Nimmo

**Affiliations:** ^1^ Department of Ocean Systems NIOZ Royal Netherlands Institute for Sea Research Yerseke The Netherlands; ^2^ Faculty of Aerospace Engineering TU Delft Delft The Netherlands; ^3^ Lunar and Planetary Laboratory University of Arizona Tucson AZ USA; ^4^ Department of Earth Sciences University of Oxford Oxford UK; ^5^ Department of Geology and Geophysics Woods Hole Oceanographic Institution Woods Hole MA USA; ^6^ Department of Earth and Planetary Sciences University of California Santa Cruz CA USA

**Keywords:** enceladus, tides, poroviscoelasticity, interior, hydrothermal

## Abstract

The inferred density of Enceladus' core, together with evidence of hydrothermal activity within the moon, suggests that the core is porous. Tidal dissipation in an unconsolidated core has been proposed as the main source of Enceladus' geological activity. However, the tidal response of its core has generally been modeled assuming it behaves viscoelastically rather than poroviscoelastically. In this work, we analyze the poroviscoelastic response to better constrain the distribution of tidal dissipation within Enceladus. A poroviscoelastic body has a different tidal response than a viscoelastic one; pressure within the pores alters the stress field and induces a Darcian porous flow. This flow represents an additional pathway for energy dissipation. Using Biot's theory of poroviscoelasticity, we develop a new framework to obtain the tidal response of a spherically symmetric, self‐gravitating moon with porous layers and apply it to Enceladus. We show that the boundary conditions at the interface of the core and overlying ocean play a key role in the tidal response. The ocean hinders the development of a large‐amplitude Darcian flow, making negligible the Darcian contribution to the dissipation budget. We therefore infer that Enceladus' core can be the source of its geological activity only if it has a low rigidity and a very low viscosity. A future mission to Enceladus could test this hypothesis by measuring the phase lags of tidally induced changes of gravitational potential and surface displacements.

## Key Points

- We present a new method to compute the tidal response of moons with porous layers
- The boundary conditions at the porous layer boundaries control the moon's response
- Tidal dissipation in Enceladus' core can only account for the moon's observed thermal output if it is in a highly deformable state

## 1 Introduction

When Voyager 1 and Voyager 2 flew by Enceladus they revealed a surprisingly young and active world (Smith et al., 1982). More than two decades later, the Cassini spacecraft discovered water plumes erupting from Enceladus' South Polar Terrain (SPT) (Porco et al., 2006), showed that the SPT radiates ∼10 GW of energy to space (Howett et al., 2011), and demonstrated that Enceladus has a global subsurface ocean (Postberg et al., 2011; Thomas et al., 2016).

Explaining the high geological activity of Enceladus remains challenging (Nimmo et al., 2018). The moon's activity is linked to diurnal tides (e.g., Yoder, 1979). Enceladus is currently in a 2 : 1 mean‐motion orbital resonance with Dione that forces its orbital eccentricity, causing time‐varying tides that periodically deform the moon. As Enceladus is not perfectly elastic, part of the tidal energy is transformed into heat, a process known as tidal heating. Energy dissipation in Enceladus is ultimately dependent on tidal dissipation in Saturn. Dissipation within the planet causes a phase‐lag in Saturn's tidal bulge; consequently, rotational energy is transferred to Enceladus and Dione where part of it is dissipated (e.g., Nimmo et al., 2018).

Astrometric observations of the Saturnian system can be used to constrain the phase‐lag of Saturn's tidal bulge and estimate the amount of energy dissipated in the moons (Meyer & Wisdom, 2007). They suggest that Enceladus is in orbital and thermal equilibrium (Fuller et al., 2016; Lainey et al., 2012). If the moon is in thermal equilibrium, ice‐shell thickness estimates combined with measurements of the SPT thermal flux can be used to obtain the total energy produced within the moon, which adds up to ∼35 GW (Hemingway et al., 2018). However, explaining where and how this much energy is dissipated within Enceladus has been problematic (e.g., Nimmo et al., 2018), giving rise to Enceladus' energy puzzle.

Enceladus' ice shell is most likely brittle and conductive, limiting the amount of heat that can be dissipated within it to about 1 GW (Beuthe, 2019; Souček et al., 2019). Frictional heating along Enceladus' tiger stripes can contribute an additional 0.1–1 GW of energy dissipation (Pleiner Sládková et al., 2021), but, overall, tidal heating in the ice shell can only account for roughly 10% of the observed SPT thermal output. Ocean tides have been proposed as an additional heating mechanism (Tyler, 2011), but they only become important if Enceladus has an orbital obliquity two orders of magnitude higher than the expected value (Chen & Nimmo, 2011); or the ocean is unrealistically thin, radially stratified or turbulent (Chen et al., 2014; Hay & Matsuyama, 2019; Matsuyama, 2014; Rekier et al., 2019; Rovira‐Navarro et al., 2019, 2020; Tyler, 2020; Wilson & Kerswell, 2018). Modeling the core as a purely solid, viscoelastic body, tidal dissipation produced within it can only account for the observed thermal output if the core has a viscosity of *η*_*c*_ < 10^13^ Pa s, much lower than that characteristic of rock. Because of this, substantial tidal dissipation in the core was first disregarded. However, Roberts (2015) and Choblet et al. (2017) recently suggested that a low enough viscosity can be attained if Enceladus' core is porous.

An Enceladan porous core is consistent with observations. The density of the core inferred from gravity data, 2.4 g cm^−3^ (e.g., Beuthe et al., 2016), is low compared to that of the minerals expected to form the bulk of the core (Choblet et al., 2017). Furthermore, the detection of salt‐rich particles (Postberg et al., 2009, 2011), silicon‐rich nanoparticles (Hsu et al., 2015) and molecular hydrogen (Waite et al., 2017) in material ejected by Enceladus' plumes suggests that the ocean interacts with the silicate core in hydrothermal systems. Taken together, these observations suggest that the moon's core is a porous, water‐saturated matrix of silicates or loosely packed rock pieces through which water can circulate.

Even though Roberts (2015) and Choblet et al. (2017) attributed Enceladus' activity to a porous core, they did not explicitly model how a porous core responds to tides. Instead, they modeled the core as a viscoelastic, rocky solid. The response of a porous, permeable, water‐saturated body to tidal forces differs from that of a pure solid. The deformation of the matrix induces a flow of water through the permeable interior, which in turns affects the response of the solid matrix and modifies the dissipation in the solid. Furthermore, the viscous flow of water through the pores adds an additional source of dissipation that may not be negligible.

While the tidal response of solid and liquid layers have been thoroughly examined (e.g., Beuthe, 2016; Chen et al., 2014; Jara‐Orué & Vermeersen, 2011; Kaula, 1964; Love, 1911; Matsuyama et al., 2018; Renaud & Henning, 2018; Rovira‐Navarro et al., 2019; Segatz et al., 1988; Tyler, 2008), the tidal response of bodies with porous layers has been subjected to much less scrutiny. Wang et al. (1999) estimated energy dissipation due to tidally induced flows in Earth's permeable seafloor and showed it to be negligible; Vance et al. (2007) applied the same approach to Enceladus' hydrothermal systems and reached a similar conclusion for the icy moon. Liao et al. (2020) developed a more complete approach based on Biot's theory of poroviscolasticity (Biot, 1941) and argued that the interaction between solid and liquid phases lead to a heat production that can easily exceed 10 GW and thus solve Enceladus' energy puzzle.

The model presented by Liao et al. (2020) included several simplifications that require further examination: (a) only the tidal response of the core was considered instead of that of the whole moon (core, ocean and ice shell); (b) the authors forced the problem via an imposed surface strain derived from viscoelastic models and only considered one component of the eccentricity tide instead of forcing the core with the complete tidal potential; (c) the authors neglected the effect of self‐gravity, the body force arising from the tidal deformation itself; and (d) they assumed that the displacement field was irrotational.

In this paper, we relax the assumptions of Liao et al. (2020) and develop a self‐consistent model to compute the tidal deformation and fluid flow of self‐gravitating bodies with porous layers (Section 2) that can be applied to Enceladus and other bodies with internal porous layers. The new approach is an extension of the standard theory of tides for self‐gravitating viscoelastic bodies (Love, 1906; Peltier, 1974; Sabadini et al., 2016; Saito, 1974; Takeuchi et al., 1962) to bodies with poroviscoelastic layers. We apply the new model to Enceladus (Section 3), and examine the circumstances under which sufficient tidal dissipation can be produced within the core to explain the thermal energy radiated by the moon. In Section 4 we conclude that Darcy dissipation is likely negligible in Enceladus' thermal budget, leaving a low‐viscosity, low‐rigidity core as the most plausible avenue for substantial tidal dissipation in the rocky core. We propose how future missions could test this hypothesis.

## 2 Methods

**Figure 1:**
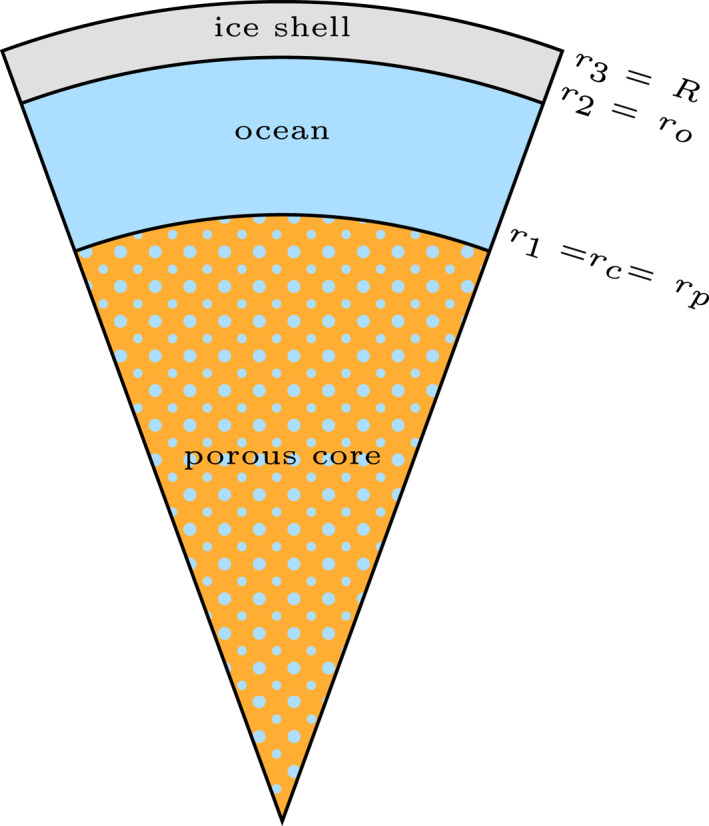
Interior structure of Enceladus consisting of three layers: a porous core, a subsurface ocean and an ice shell. The porous core boundary is assumed to be permeable.

Our aim is to obtain the linear, periodic, tidal response of a body with internal porous layers to a tidal perturbation. We assume the moon is composed of *N* spherically symmetric layers of uniform properties. The boundary between a layer *i* and *i* + 1 is at radius *r*_*i*_. We consider layers that are either purely liquid, purely solid, or a contiguous solid matrix with a permeable network of liquid‐filled pores. We use a viscoelastic model of the solid. The model can include as many layers as required to approximate the interior structure of the moon under consideration. Figure 1 shows the interior structure that is thought to be valid for Enceladus, consisting of an icy shell, a subsurface ocean and a porous core.

Liao et al. (2020) applied Biot's theory of poroviscoelasticity to the tidal problem. However, the present work is the first derivation of a self‐consistent model of body tides in porous media; we therefore provide a detailed formulation of the problem, highlight key assumptions, and explain how it differs from the standard tidal theory for viscoelastic solids and the work of Liao et al. (2020). To this end, we start by presenting the governing equations for porous media in detail, show how they reduce to those of a pure solid, and discuss how internal liquid layers are modeled (Section 2.1); then we introduce the tidal potential and boundary conditions (Section 2.2); and, finally, sketch the solution method (Section 2.3), leaving full details of the mathematical formulation to the Appendices.

### 2.1 Governing Equations

We use the volume‐averaged mass and momentum conservation equations for a parcel of the moon. The parcel contains a solid and a liquid phase of densities *ρ*_*s*_ and *ρ*_*l*_ and volumes *V*_*s*_ and *V*_*l*_, respectively. The parcel is at least one order of magnitude bigger than the typical grain size. This way, we can use continuum mechanics rather than explicitly modeling the microphysical interactions of grains within the parcel. Thus, the variables that follow should be understood as averages. The porosity of the parcel is simply defined as the ratio between the liquid (*V*_*l*_) and total (*V*) volumes:

(1) $$\Phi =\frac{V_{l}}{V}.$$

The mass conservation equations for the liquid and solid phases can be written as (e.g., Ganesan & Poirier, 1990)

(2a) $$\frac{\partial \Phi \rho_{l}}{\partial t}+\nabla \cdot \left[\Phi \rho_{l}v_{l}\right]=0,$$

(2b) $$\frac{\partial \left(1-\Phi \right)\rho_{s}}{\partial t}+\nabla \cdot \left[\left(1-\Phi \right)\rho_{s}v_{s}\right]=0.$$

Equations 2a and 2b can be added to obtain

(3) $$\frac{\partial \rho }{\partial t}+\nabla \cdot \left[\rho_{l}q+\rho v_{s}\right]=0.$$

*ρ* is the bulk density, *ρ* = Φ*ρ*_*l*_ + (1 − Φ)*ρ*_*s*_; and ***q*** is the segregation flux given by the relative velocity of the liquid phase with respect to the solid phase, ***q*** = Φ(***v***_*l*_ − ***v***_*s*_). We additionally introduce the variation of fluid content, defined as the amount of liquid entering the solid frame per unit of the solid frame (Cheng, 2016),

(4) $$\zeta =\Phi \nabla \cdot \left(u_{s}-u_{l}\right),$$

with ***u*** being a displacement vector.

The momentum equation in a frame rotating with the moon’s angular velocity (*ω*) is given by (McKenzie, 1984)

(5a) $$\Phi \rho_{l}\left(\frac{Dv_{l}}{Dt}+2\omega \times v_{l}\right)=\nabla \cdot \Phi \sigma_{l}-\Phi \rho_{l}\nabla \phi -F,$$

(5b) $$(1-\Phi )\rho_{s}\left(\frac{Dv_{s}}{Dt}+2\omega \times v_{s}\right)=\nabla \cdot \left(1-\Phi \right)\sigma_{s}-\left(1-\Phi \right)\rho_{s}\nabla \phi +F.$$

***σ*** is the stress tensor, *ϕ* is a potential that includes gravitational forces and the centrifugal force. ***F*** is an interaction force between the solid and liquid phases given by

(6) $$F=\Phi \frac{\eta_{l}}{\kappa }q-p\nabla \Phi .$$

*p* is the pore pressure and *η*_*l*_ is the liquid viscosity. *κ* is the matrix permeability, which depends on the geometry of the solid matrix. For a solid matrix made of uniform, spherical grains of size *d*_*g*_, a commonly used expression is the Kozeny‐Carman law (Carman, 1997; Kaviany, 1995):

(7) $$\kappa =\frac{\Phi^{3}}{180{\left(1-\Phi \right)}^{2}}d_{g}^{2}$$

The inertial terms in the momentum equations (Equation (5a), (5b)) can be neglected. For the solid phase, the high viscosity and the long period of tidal forces as compared to seismic waves imply that the solid is in quasi‐equilibrium. In a porous medium, the interaction force is generally larger than inertial terms in Equation 5b, which in turn results in a small Reynolds number. Summing the two momentum equations, an equation for the bulk or total stress is obtained

(8) ∇⋅σ−ρ∇ϕ=0,

with the total stress tensor being

(9) $$\sigma =\left(1-\Phi \right)\sigma_{s}+\Phi \sigma_{l}.$$

We assume that deviatoric stresses in the liquid fluctuate on the pore scale and hence they volume‐average to zero, except for their contribution to the interaction force (Equation 6). The stress tensor of the liquid phase is thus isotropic and given by the pore pressure,

(10) $$\sigma_{l}=-pI,$$

with ***I*** being the identity matrix. Using the interaction force expression, the liquid phase momentum equation reduces to Darcy's law,

(11) $$q=-\frac{\kappa }{\eta_{l}}\left(\nabla p+\rho_{l}\nabla \phi \right).$$

Note that if the porosity is zero, we recover the mass and momentum conservation equations for a solid.

A constitutive equation relating the stress tensor and pore pressure to kinematic variables is needed. We define the strain tensor as

(12) $$\epsilon =\frac{1}{2}\left[\nabla u+{\left(\nabla u\right)}^{T}\right],$$

where ***u*** is the volume average of the solid displacement (***u***_*s*_). The stress and strain tensors can be split into a mean and a deviatoric component:

(13a) $$\sigma =-PI+\sigma^{d},$$

(13b) $$\epsilon =\epsilon^{M}I+\epsilon^{d},$$

with *ϵ* ^*M*^ = tr(***ϵ***)/3 and *P* = −tr(***σ***)/3; tensile stress is taken to be positive.

In a compressible poroviscoelastic solid, deformation is associated with an effective stress ***σ*** ′ (Biot, 1941; Cheng, 2016),

(14) $$\sigma^{\prime }=\sigma +\alpha pI.$$

*α* is Biot's constant, the meaning of which will become evident later. We consider that the material is viscoelastic. Different rheological laws can be used to consider the behavior of a viscoelastic material (e.g., Renaud & Henning, 2018). We consider the Maxwell model, in which case effective stress and strain are related as

(15) $$\frac{d\sigma^{\prime }}{dt}+\frac{\mu }{\eta }\sigma^{\prime }-\frac{1}{3}\frac{\mu }{\eta }tr\left(\sigma^{\prime }\right)I=2\mu \frac{d\epsilon }{dt}\left(K-\frac{2}{3}\mu \right)\frac{d\;tr\left(\epsilon \right)}{dt}I,$$

or, in terms of total stress,

(16) $$\frac{d\sigma }{dt}+\frac{\mu }{\eta }\sigma -\frac{1}{3}\frac{\mu }{\eta }tr\left(\sigma \right)I=2\mu \frac{d\epsilon }{dt}+\left(K-\frac{2}{3}\mu \right)\frac{d\;tr\left(\epsilon \right)}{dt}I-\alpha \frac{dp}{dt}I.$$

Here, *μ* and *η* are the shear modulus and viscosity of the two‐phase aggregate, respectively. The Maxwell model does not capture the anelastic behavior of ices and silicates, which can become especially important when the forcing period is much smaller than the Maxwell time *η*/*μ* (Efroimsky, 2012). It is for this reason that recent studies have considered the Andrade rheological model (Andrade & Trouton, 1910) for ices (e.g., Castillo‐Rogez et al., 2011; Gevorgyan et al., 2020; Rambaux et al., 2010; Rhoden & Walker, 2022; Shoji et al., 2013) and silicates (e.g., Bierson & Nimmo, 2016; Efroimsky, 2012; Renaud & Henning, 2018; Rovira‐Navarro et al., 2021; Walterová & Běhounková, 2017). In Appendix F we discuss how more complex rheology models can be incorporated into our theory and demonstrate this using the Andrade model.

One further constitutive equation relates the pore pressure with the isotropic strain and the variation of fluid content as (Cheng, 2016),

(17) $$p=\frac{K_{u}-K}{\alpha }tr\left(\epsilon \right)+\frac{K_{u}-K}{\alpha^{2}}\zeta ,$$

where *K*_*u*_ and *K* are the bulk modulus of the material in undrained (*ζ* = 0) and drained (*p* = 0) conditions, respectively. These are effective properties of the two‐phase medium; the drained modulus depends on the mechanical properties of the solid matrix; in contrast, the undrained modulus depends on both the properties of the liquid and the solid phases. If the material is microscopically homogeneous and isotropic, *K*, *K*_*u*_ and *α* can be obtained using the bulk modulus of the solid *K*_*s*_ and liquid *K*_*l*_ phases, and the *bulk modulus of porosity* *K*_Φ_, which measures the resistance to grain rearrangement, (e.g., Cheng, 2016),

(18a) $$\alpha =\frac{1+\Phi {\left(1-\Phi \right)}^{2}K_{\Phi }/K_{s}}{1+{\left(1-\Phi \right)}^{2}K_{\Phi }/K_{s},}$$

(18b) $$K=\frac{{\left(1-\Phi \right)}^{3}K_{\Phi }/K_{s}}{1+{\left(1-\Phi \right)}^{2}K_{\Phi }/K_{s}}K_{s}=\left(1-\alpha \right)K_{s},$$

(18c) $$K_{u}=K+\frac{K_{l}{\left(K_{s}-K\right)}^{2}}{K_{l}\left(K_{s}-K\right)+\Phi K_{s}\left(K_{s}-K_{l}\right)}.$$

With this definition, it becomes apparent that the Biot parameter *α* relates the resistance to compression of the solid constituent and the porous matrix. A strong porous matrix (e.g., spherical holes) has a small *α* while an easily deformable matrix (i.e., slit or crack‐shaped pore spaces) has an *α* close to 1. If the solid is much less compressible than the frame, *K*_Φ_/*K*_*s*_ → 0 and therefore *α* → 1.

The previous set of constitutive equations define a material that, upon a stress perturbation, exhibits an elastic response and viscous creep. Moreover, a perturbation produces a pore pressure field that drives Darcian porous flow. Viscous creep and Darcian flow result in energy dissipation. The rate of volumetric tidal dissipation averaged over a tidal cycle due to these two processes is (e.g., Liao et al., 2020)

(19a) $${\dot{E}}_{v,solid}=\frac{1}{T}\int_{0}^{T}\left(\sigma :\frac{\partial \epsilon }{\partial t}+p\frac{\partial \zeta }{\partial t}\right)dt,$$

(19b) $${\dot{E}}_{v,liquid}=\frac{1}{T}\int_{0}^{T}\frac{\eta_{l}}{\kappa }\left(q\cdot q\right)dt,$$

respectively.

If the body has an internal liquid layer (i.e., ocean), alternate equations are required for that layer. We assume internal liquid layers are inviscid, incompressible and in hydrostatic equilibrium and the radial displacements follow equipotential surfaces,

(20) $$u\cdot e_{r}=-\phi /g,$$

except at solid‐liquid interfaces, where this might be hindered (Jara‐Orué & Vermeersen, 2011). ***e***_*r*_ is the radial unit vector, and *g* the gravitational acceleration. Under these assumptions, surface (e.g., Hay & Matsuyama, 2017; Matsuyama, 2014; Rovira‐Navarro et al., 2020; Tyler, 2011) and internal waves (Rekier et al., 2019; Rovira‐Navarro et al., 2019) are excluded from the solution.

In all layers, the gravitational potential of the body can be computed using Poisson's equation,

(21) $$\nabla^{2}\phi =4\pi G\rho .$$

### 2.2 Tidal Forcing and Boundary Conditions

We consider a synchronously rotating moon of radius *R* with an orbital frequency *ω* and eccentricity *e*. As the obliquity of Enceladus is expected to be very small (Chen & Nimmo, 2011), we focus on eccentricity tides and ignore obliquity tides. The tidal potential at a point with co‐latitude and longitude *θ*, *φ* located at radial distance *r* from the center of the moon is given by (e.g., Jara‐Orué & Vermeersen, 2011; Kaula, 1964)

(22) $$\begin{array}{lll} \phi^{T}\left(r,\theta ,\varphi \right) & = & {\left(\omega R\right)}^{2}e{\left(\frac{r}{R}\right)}^{2}Re\left\{\left(3\sqrt{\frac{\pi }{5}}Y_{2}^{0}\left(\theta ,\varphi \right)\right.\right.\\ & & \left.\left.-3\sqrt{\frac{3\pi }{5}}Y_{2}^{2}\left(\theta ,\varphi \right)+4\sqrt{\frac{3\pi }{5}}iY_{2}^{-2}\left(\theta ,\varphi \right)\right)\exp\left(i\omega t\right)\right\}+O\left(e^{2}\right). \end{array}$$

$Y_{l}^{m}$ are normalized, real spherical harmonics of degree *l* and order *m* (Equation A2).

To solve the previous set of equations, boundary conditions at the moon's surface are required. The normal and shear stress at the surface are zero,

(23) $$\sigma_{rr}\left(R\right)=\sigma_{r\theta }\left(R\right)=\sigma_{r\phi }\left(R\right)=0.$$

the potential, *ϕ*, is continuous at the surface but its gradient is not. Using Poisson's equation and applying Gauss' theorem for an infinitesimal control volume surrounding the surface layer, we find

(24) $$\int_{S}\nabla \phi \cdot e_{r}dS=4\pi G\int_{S}\int_{R-\delta }^{R+\delta }\rho drdS.$$

We note that Liao et al. (2020) considered only the zonal component of the tidal potential (*m* = 0). Furthermore, the tidal forcing was imposed via a prescribed strain at the surface of the core instead of via the tidal potential *ϕ* ^*T*^ as explained here, and the no‐stress boundary conditions (Equation 23) were not used. The distinct effect of each of these boundary conditions will be explored in Section 3.

Additional boundary conditions must be prescribed at internal boundaries. For the core–ocean and ocean–ice shell interfaces, we use the boundary conditions discussed in Jara‐Orué and Vermeersen (2011) and given in Appendix A. Nevertheless, an additional boundary condition should be provided at the porous layer interface (*r*_*p*_). Two different boundary conditions can be considered: no radial Darcy flux

(25) $$q\left(r_{p}\right)\cdot e_{r}=0,$$

or force balance and continuity of fluid pressure. In the latter case, the ocean pressure at the core surface is balanced by the radial component of the stress tensor and the pore pressure equals the ocean pressure,

(26) $$\sigma_{rr}\left(r_{p}\right)=-P_{\mathrm{ocean}},\;p\left(r_{p}\right)=P_{\mathrm{ocean}},$$

which implies *p*(*r*_*p*_) + *σ*_*rr*_(*r*_*p*_) = 0. For Enceladus, we consider that the core–ocean boundary is permeable and thus use the second boundary condition.

### 2.3 Perturbation Theory and Solution Method

To make the analysis tractable, we linearize the equations of motion using perturbation theory. We split the density, stress tensor and potential force into a background and a perturbed component. The background component corresponds to the pre‐stressed, hydrostatic state arising from the self‐gravity of the body; the perturbed component is the result of the time‐dependent tidal potential,

(27a) $$\sigma =-P_{0}I+\sigma^{\Delta },$$

(27b) $$p=p_{0}+p^{\Delta },$$

(27c) $$\rho =\rho_{0}+\rho^{\Delta },$$

(27d) $$\rho_{l}=\rho_{l,0}+\rho_{l}^{\Delta },$$

(27e) $$\phi =\phi_{0}+\phi^{\Delta }.$$

Here, *ϕ*_0_ is the gravitational potential of the unperturbed body and *ϕ* ^Δ^ includes both the perturbing tidal potential and the potential arising from self‐gravitation of the perturbed body. In the unperturbed state, Equations 8 and 11 are given by:

(28a) $$\nabla P_{0}+\rho_{0}\nabla \phi_{0}=0,$$

(28b) $$\nabla p_{0}+\rho_{l,0}\nabla \phi_{0}=0,$$

with ∇*ϕ*_0_ = *g* ***e***_*r*_.

Using the previous definitions and linearizing by assuming that the products of perturbation variables are negligible, the momentum equations can be written as:

(29a) $$\nabla \cdot \sigma^{\Delta }-\nabla \left(\rho_{0}gu\cdot e_{r}\right)-\rho_{0}\nabla \phi^{\Delta }-\rho^{\Delta }ge_{r}=0,$$

(29b) $$q=-\frac{\kappa }{\eta_{l}}\left(\nabla p^{\Delta }+\rho_{l,0}\nabla \phi^{\Delta }+g\rho_{l}^{\Delta }e_{r}\right).$$

The mass conservation equation can be obtained by linearizing Equation 3 and assuming that under small displacements the Lagrangian and Eulerian derivatives are approximately equal,

(30) $$\frac{\rho^{\Delta }}{\rho_{0}}=-\nabla \cdot u+\frac{\rho_{l,0}}{\rho_{0}}\zeta .$$

Similarly, using the definition of the variation in liquid content (Equation 4), and the segregation flux, we obtain:

(31) $$\frac{\partial \zeta }{\partial t}=-\nabla \cdot q.$$

Finally, the density change of the liquid phase can be obtained using the definition of the liquid bulk modulus,

(32) $$\frac{\rho_{l}^{\Delta }}{\rho_{l,0}}=\frac{p^{\Delta }}{K_{l}}.$$

The perturbed gravitational potential is obtained by solving the linearized Poisson's equation,

(33) $$\nabla^{2}\phi^{\Delta }=4\pi G\rho^{\Delta }.$$

Equations (29a), (29b), (30), (31), (32), (33) reduce to those used in Liao et al. (2020) if the solid and liquid phases are assumed to be massless (*ρ*_0_, *ρ*_*l*,0_ = 0). If the layer is purely solid (*Φ* = 0, *α* = 0), we recover the classic equations used for a viscoelastic solid (Sabadini et al., 2016).

To obtain the tidal response of the body, the momentum Equation (29a), (29b), mass conservation Equations 30 and 31, and Poisson's Equation 33 together with constitutive Equations 16 and 17 should be solved under appropriate boundary conditions (Section 2.2). As the tidal forcing is periodic, we solve the equations of motion in the Fourier domain. We assume a solution proportional to exp(*iωt*) and transform the previous set of equations to the Fourier domain. Because of the symmetry of the problem, we solve the previous set of equations using spherical harmonics. We obtain stress and strain tensors, the pore pressure and Darcy flow, and we compute tidal dissipation in the solid and liquid phases using Equation (19a), (19b). Further details can be found in Appendices A, B, C, D.

## 3 Application to Enceladus

To understand how the predictions of this model differ from the previous treatment of Liao et al. (2020) we consider three different cases: (a) Enceladus' core forced via a prescribed surface strain as in Liao et al. (2020); (b) Enceladus' core with a free surface and forced with the tidal potential; and (c) a complete model of Enceladus consisting of a porous core, an ocean, and an ice shell, forced with the tidal potential. We begin by examining the simpler cases 1 and 2 to illustrate the effect of the boundary conditions used by Liao et al. (2020) (Section 3.1) and then move to the more complex, multilayered model to show how the ocean and ice shell affect the core's tidal response (Section 3.2).

We assume a core density of 2.4 g cm^−3^ consistent with gravity observations (Beuthe et al., 2016). Choblet et al. (2017) obtained a core porosity of 20–30% for realistic core compositions; we use a value of 20%. For a consolidated silicate core, the shear modulus is ∼1–10 GPa and the viscosity is ∼10^20^ Pa s or higher at low homologous temperature. However, if the core is unconsolidated, it can become weaker and the shear modulus and viscosity can be orders of magnitude lower than the typical values of silicates (Choblet et al., 2017; Goldreich & Sari, 2009; Nimmo et al., 2018). The parameters used are summarized in Table 1.

**Table 1:** Enceladus Physical and Mechanical Properties

Quantity	Symbol	Value	Units
Surface radius	*R*	252.1	km
Mass	*M*	1.08 ⋅ 10^20^	kg
Ocean thickness^a^	*h*_*ocean*_	38	km
Ice shell thickness^a^	*h*_*ice*_	23	km
Average core's density^a^	*ρ*_*core*_	2422	kg m^−3^
Ocean's density	*ρ*_*ocean*_	1000	kg m^−3^
Ice viscosity^b^	*η*_*ice*_	1 ⋅ 10^18^	Pa s
Ice shear modulus^b^	*μ*_*ice*_	3.3	GPa
Ice bulk modulus	*K*_*ice*_	33	GPa
Core shear modulus	*μ*_*s*_	0.01–10	GPa
Core solid phase bulk modulus^c^	*K*_*s*_	10	GPa
Core viscosity	*η*_*s*_	10^10^–10^20^	Pa s
Biot's constant	*α*	0–1	−
Water viscosity^c^	*η*_*l*_	1.9 ⋅ 10^−3^	Pa s
Water bulk modulus^c^	*K*_*l*_	2.2	GPa
Core permeability^c^	*κ*	10^−8^ − 10^−4^	m^2^
Core porosity	Φ	0.2	‐
Eccentricity	*e*	0.0047	‐
Orbital Period	*T*	33	h

^a^ Beuthe et al. (2016).

^b^ Hussmann and Spohn (2004).

^c^ Liao et al. (2020).

### 3.1 Core‐Only Model

Liao et al. (2020) studied the response of Enceladus' core to a prescribed radial strain imposed at the core's surface (in this section, *R* = *r*_*c*_). The strain was given by a degree‐2 order‐0 field of the form: $\epsilon_{rr}\left(R,\theta ,\varphi \right)=\epsilon \left(R\right)Y_{2}^{0}$. The amplitude of the strain was estimated from the theory of viscoelastic tides (Murray & Dermott, 2000). For a homogeneous body in which rigidity dominates over self‐gravity, the maximum radial strain attained at the poles is $\epsilon_{rr}\left(R,0{}^{\circ}\right)=\frac{9}{4\pi }\frac{\omega^{2}}{\rho G}e\frac{5}{3}|k_{2}|$, where $k_{2}=\frac{3/2}{1+19\hat{\mu }/2\rho gR}$ is the gravitational potential Love number and $\hat{\mu }$ is the complex rigidity. Additionally, Liao et al. (2020) assumed the displacement field to be irrotational, ∇ × ***u*** = 0, and considered the solid and liquid to be massless (*ρ*, *ρ*_*l*_) = 0. For Case 1 we make the same assumptions.

Liao et al. (2020) found that for Biot parameter *α* → 1, tidal dissipation is enhanced as compared to standard viscoelastic models. In what follows, we assess whether this still holds when the core is forced via the tidal potential rather than via a prescribed surface strain. We first take a fixed core permeability (*κ* = 10^−8^ m^2^), and compute tidal dissipation for different values of core viscosity and Biot parameter *α*. Changing *α* for a given porosity is equivalent to changing the ratio between the bulk modulus of porosity and the solid bulk modulus (Equation (18a), (18b)), as *α* approaches 1 the porous matrix becomes more compressible. Afterward, we study the role of the core's permeability. While we keep the porosity fixed, we note that variations in porosity between 0.2 and 0.3 result in changes of *α* and *κ* much smaller than the ranges explored below (Equations 7 and (18a), (18b), (18c)).

**Figure 2:**
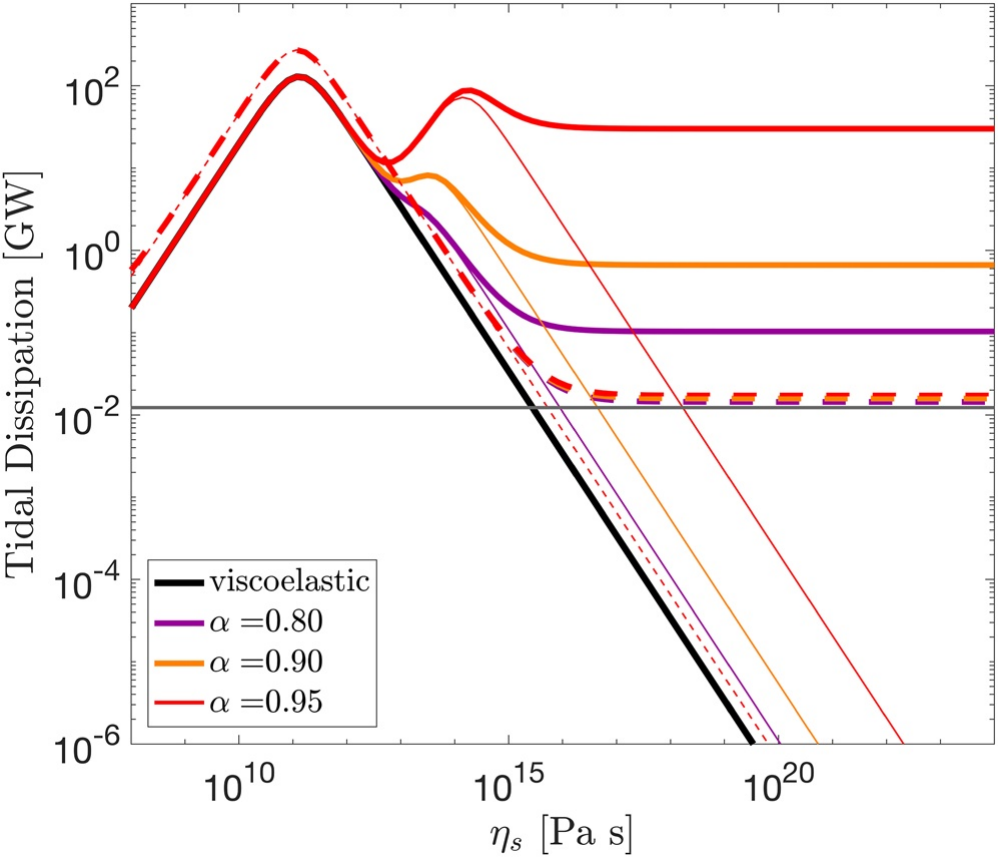
Tidal dissipation in Enceladus' core when forced with a prescribed surface strain (solid lines) or the tidal potential (dashed line) for different values of Biot coefficient *α*. The thin lines indicate tidal dissipation in the solid layer, the gray line shows the amount of Darcy dissipation for incompressible solid and liquid phases obtained using Equation (7), (18a), (18b), (18c).

**Figure 3:**
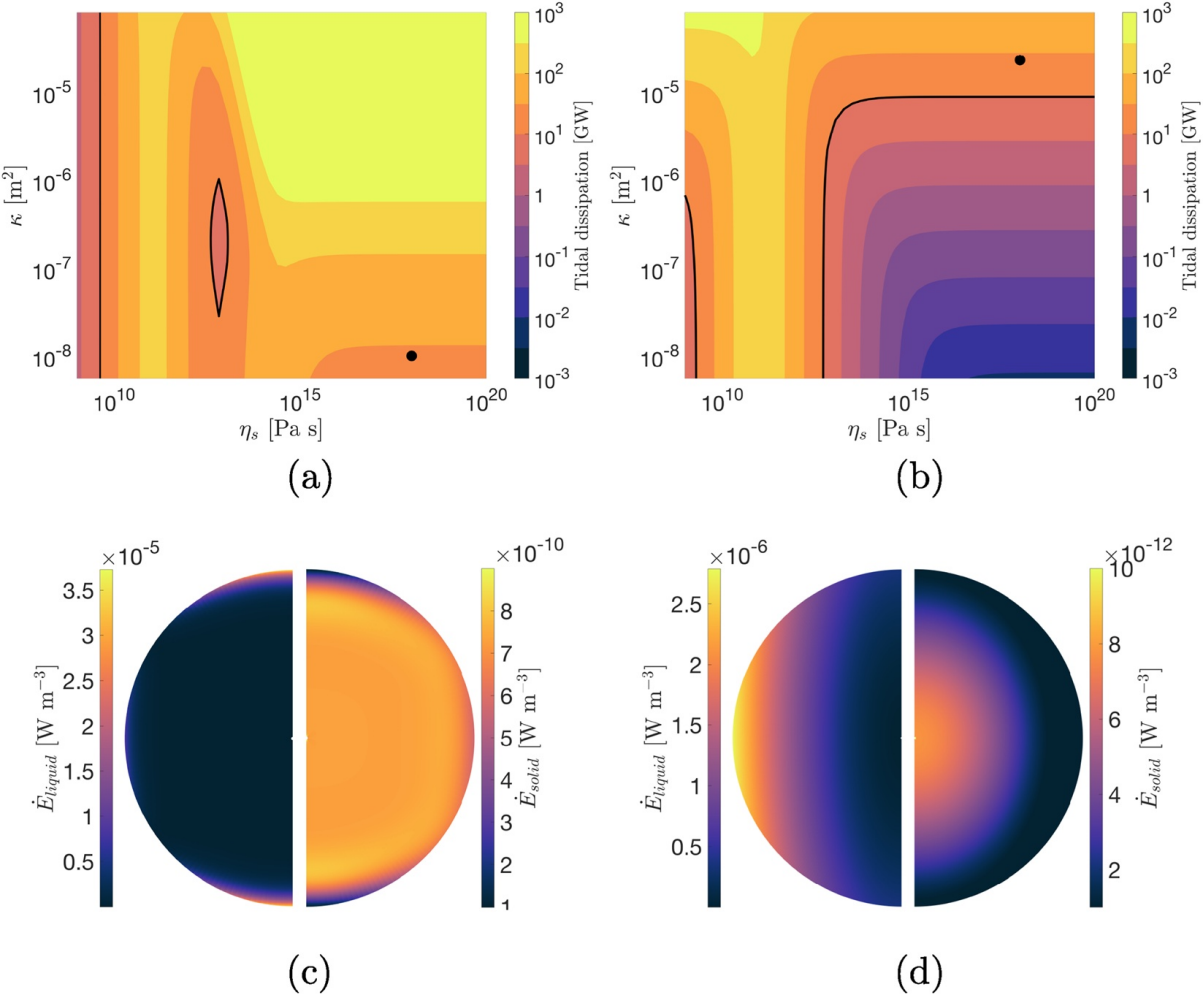
Total tidal dissipation in an Enceladan core with a free surface for various values of core viscosity and permeability. In (a) the core is forced via a prescribed strain field of order degree 2 and order 0, in (b) via the tidal potential. The contour for $\dot{E}=10$ GW is indicated in both plots. (c) and (d) show tidal dissipation in the liquid and solid phases for a meridional cut at longitude 0° for the two points indicated in (a) and (b). Both points have a viscosity of 10^18^ Pa s and result in the same amount of tidal dissipation (28 GW). For all cases we assume *μ* = 1 GPa, *K*_*s*_ = 10 GPa, *K*_*l*_ = 2.2 GPa and *α* = 0.95.

Figure 2 shows total tidal dissipation in the core for different values of Biot parameter *α* and core viscosity *η*_*s*_ for cases 1 and 2. For Case 1, we reproduce the results of Liao et al. (2020). For *α* = 0.95 tidal dissipation features two peaks, one at a core viscosity of ∼10^11^ Pa s, also characteristic of the viscoelastic response, and another one attained at higher core viscosity ∼10^15^ Pa s, only characteristic of the poroviscoelastic model. Around the two peaks, most of the energy dissipation occurs in the solid phase, as shown by the thin lines. As core viscosity increases further, dissipation in the solid decreases but the total dissipation remains high due to Darcy dissipation. This dissipation occurs in a shallow layer close to the core's surface (Figure 3c), where a strong pressure gradient develops that drives flows of up to 2 × 10^−5^ m s^−1^. The second dissipation peak is the result of the compressibility of the porous matrix. As *α* → 1, the drained bulk modulus decreases (Equation (7), (18a), (18b), (18c)), the second peak becomes more prominent, and Darcy dissipation also increases.

For Case 2, forcing by the tidal potential, the second dissipation peak is not present (heavy dashed line in Figure 2). Tidal dissipation reaches its maximum at the same core viscosity as in the non‐porous, viscoelastic case and then decreases as viscosity increase until Darcy dissipation becomes dominant. However, as opposed to (1), the amount of heat resulting from Darcy dissipation is less sensitive to the porous‐matrix compressibility and, more importantly, it is severely reduced. The prominent pressure gradients characteristic of Case 1 do not develop and the maximum flow velocities attained are reduced by two orders of magnitude. This suggests that explaining Enceladus' thermal budget in terms of poroviscoelastic dissipation may be more problematic. However, a highly permeable core may mitigate this to some extent.

The permeability dictates how easily water can flow through the core. Figures 3a and 3b show the total amount of internal heat production for cases 1 and 2 for different values of core permeability. As before, for both cases we observe the high dissipation band characteristic of a viscoelastic core with a low viscosity (∼10^11^ Pa s). As core viscosity increases Darcian dissipation becomes dominant and the total tidal dissipation becomes independent of core viscosity. In this regime, dissipation increases with permeability and the flow velocity is controlled by the dimensionless number *Ω*_*D*_ = *ωR* ^2^ *η*_*l*_/*κμ*, which can be understood as a ratio between the timescale of Darcy flow (*R* ^2^ *η*_*l*_/*κμ*) and of the tidal perturbation (1/*ω*). When *Ω*_*D*_ ≪ 1, high flow velocities are attained ($q\propto \Omega_{D}^{-1}$, Equation 11) which in turn results in high values of tidal dissipation (${\dot{E}}_{\mathrm{liquid}}\propto \Omega_{D}^{-1}$, Equation (7), (18a), (18b), (18c)). We note that when *Ω*_*D*_ becomes very high, some of the terms in the equations (e.g., the term *A*_87_ in Equation (7), (18a), (18b), (18c)) can become very large, causing numerical problems. This limits the lowest value of permeability we can attain under our current formulation to ∼10^−9^ m^2^. Nevertheless, we derive an analytical expression for Darcian dissipation in the limit of an incompressible porous matrix and liquid (Appendix E) that presents good agreement with the numerical results (Figure 2) and shows that Darcian dissipation can be expected to further decrease for lower permeability values.

While dissipation increases with permeability in both cases, Darcy dissipation in the tidally forced case is lower than in the case where a surface strain is prescribed. In Case 1) a high surface stress follows from the imposed strain, producing a large gradient in pore pressure and driving high‐amplitude Darcian flow; in contrast, in Case 2 the no‐stress boundary conditions prevent this from occurring. To produce an amount of heat similar to that observed, a permeability of *κ* > 10^−5^ m^2^ is required (Figure 3b). These are high permeability values compared to the permeability of Earth's hydrothermal systems, which can reach values of about 10^−8^ m^2^ (Lauer et al., 2018). However, it is possible that Enceladus' core does not resemble such a system, but is instead akin to an unconsolidated rubble pile. In that case, Enceladus' core would be made up of loosely packed material through which water can easily circulate.

If Enceladus' core has a porosity of ∼0.2–0.3, a permeability of ∼10^−5^ m^2^ requires grain sizes of about ∼10–50 cm (Equation 7). This blocky structure could be the relic of a violent formation process such as Enceladus forming after a series of collisions of a previous generation of moons (Asphaug & Reufer, 2013; Ćuk et al., 2016).

### 3.2 Multi‐Layered Model

We now move to the more realistic multi‐layered model consisting of a porous core, a subsurface ocean and an ice shell (Case 3). Although Enceladus' ice shell is of variable thickness (e.g., Beuthe et al., 2016; Hemingway & Mittal, 2019; Čadek et al., 2016), we consider an ice shell of constant thickness equal to its average value. For the core radius, core density, and ice and ocean thicknesses we use values in agreement with gravity, shape and libration data (Beuthe et al., 2016). We keep these values and the rheological properties of the ice constant and vary the properties of Enceladus' core (Table 1).

**Figure 4:**
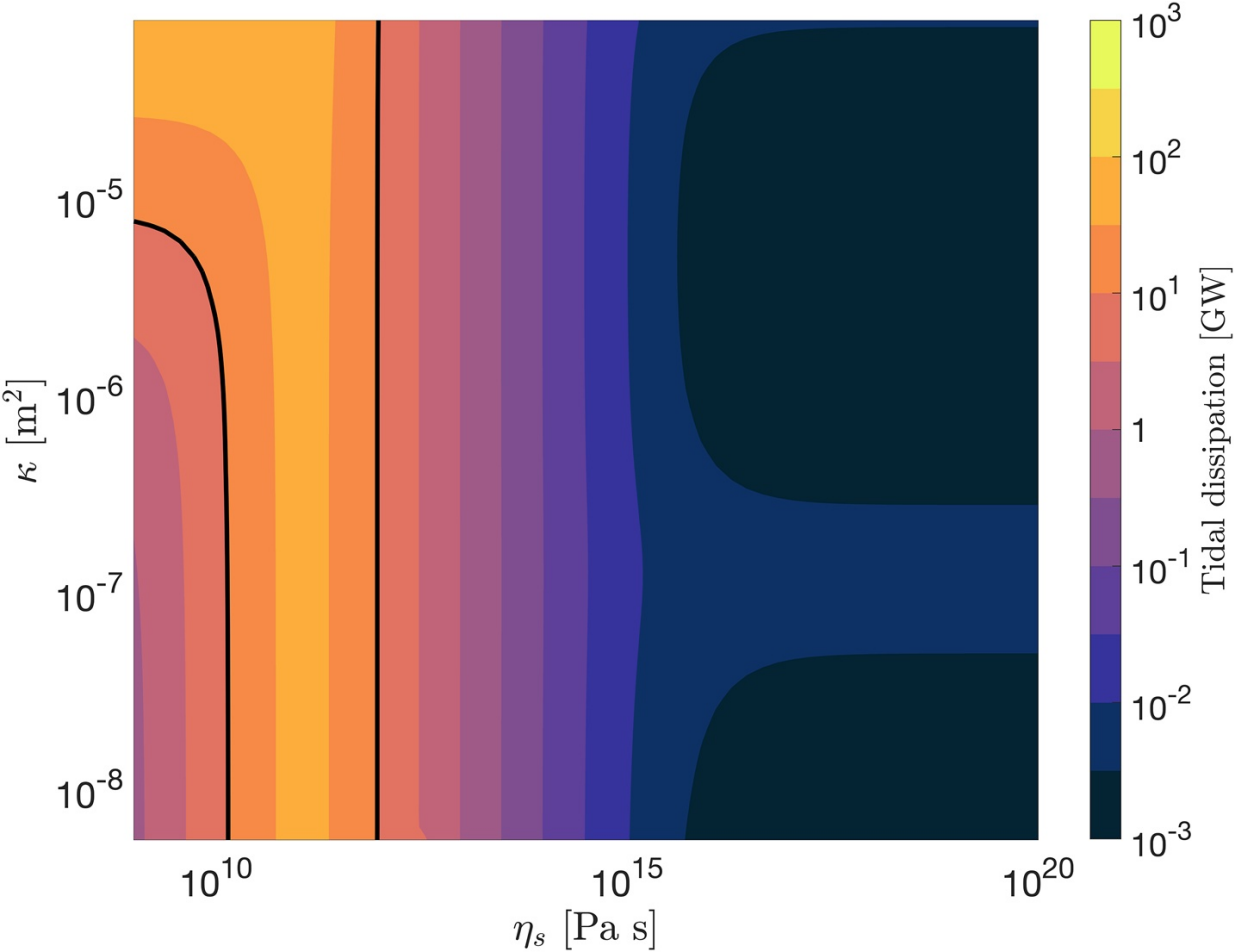
Total tidal dissipation in Enceladus' core for different values of core viscosity and permeability. We assume *μ* = 1 GPa, *K*_*s*_ = 10 GPa, *K*_*l*_ = 2.2 GPa and *α* = 0.95. The contour for $\dot{E}=10$ GW is indicated.

As in the previous cases, we compute the tidal response of the moon for different values of core viscosity and permeability. The results are shown in Figure 4; they demonstrate that the presence of an ocean reduces the tidal response of the core. As in Cases 1 and 2, we find a peak in tidal dissipation for a core viscosity of ∼10^11^ Pa s. Around this value, tidal heating is compatible with Enceladus' thermal output. However, the high‐dissipation band is narrower than for Cases 1 and 2—the presence of an ocean and an ice crust reduces the tidal deformation of the core (Beuthe, 2015). Most importantly, the amount of Darcian dissipation is drastically reduced.

As opposed to Case 2, Darcian dissipation is small even for a highly permeable core. The reduction in Darcian dissipation is due to the presence of the overlying ocean and ice shell. They impose a non‐zero pressure at the core surface that largely balances the forcing of the tidal potential. The Darcian flow *q* is driven by the modified pressure p* = *p* ^Δ^ + *ρ*_*l*,0_ *ϕ* ^Δ^ (Equation (29a), (29b)). Without an ocean, the pressure at the surface *p* ^Δ^ is 0 and the tidal force drives the Darcian flow, p* = *ρ*_*l*,0_ *ϕ* ^Δ^. In contrast, if there is an overlying ocean and ice shell, the pressure at the core–ocean boundary is not zero. To understand this, consider a core that is just covered by an ocean. Under our assumptions, the ocean surface follows the equilibrium tide −*ϕ* ^Δ^/*g* while the core–ocean boundary has a radial displacement of *u*. As a result, the pressure perturbation at the core–ocean boundary is *p* ^Δ^ = *ρ*_*l*,0_ *g*(−*u* − *ϕ* ^Δ^/*g*). This ocean pressure partially compensates the driving tidal potential, leading to a modified pressure p* proportional to the the core radial displacement −*ρ*_*l*,0_ *gu*. Writing this in terms of *h*_2_, the radial displacement Love number, p* = *ρ*_*l*,0_ *h*_2_ *ϕ* ^Δ^. Because of its rigidity, the radial displacement of the core is generally much smaller than the equilibrium tide (*h*_2_ ≪ 1), explaining why Darcy dissipation is greatly reduced when considering an ocean‐covered core.

The presence of an ice shell above the ocean inhibits the ocean surface from following the equilibrium tide by imposing a pressure load. If the ice shell is completely rigid (no surface displacements), the pressure load compensates the equilibrium tide and is given by −*ρ*_*l*,0_ *ϕ* ^Δ^. Hence the ice shell imposes a pressure equivalent to that imposed by the water column in an ice‐free ocean, leading to the same reduction in effective pressure at the core. Appendix E provides analytical solutions for an incompressible core with and without a free surface that further demonstrate the role of the pressure at the core–ocean boundary.

### 3.3 An Unconsolidated Core

In light of the reduced tidal dissipation in Enceladus' porous core in comparison to the findings of Liao et al. (2020), we reconsider the low viscosity band where dissipation is enhanced. In all cases, we found that tidal dissipation peaks at ∼10^11^ Pa s. Close to this viscosity value, the amount of heat dissipated within the core is compatible with Enceladus' observed thermal output. Such a low viscosity is incompatible with the viscosity characteristics of silicates (Roberts & Nimmo, 2008). However, Choblet et al. (2017) proposed that low effective viscosity values can be attained if Enceladus' core is unconsolidated. If this is the case, friction between grains can give rise to substantial dissipation. We next reconsider this hypothesis and discuss its plausibility and remaining unknowns.

If strains are small, a granular material essentially behaves as a monolith. The grains deform elastically and stresses are transmitted at the grain boundaries. Some viscous deformation can also occur mainly due to diffusion creep. Under higher strains, grain–grain sliding becomes important and, combined with inter‐granular friction, it can result in enhanced energy dissipation (Lambe & Whitman, 1969). Instead of using *μ* and *η*_*s*_ to characterize the rheology, a granular material is normally characterized in terms of an effective shear modulus (*μ*_*eff*_) and a damping coefficient (υ) (e.g., Choblet et al., 2017; Seed et al., 1986). These two variables are related to the shear modulus and the solid viscosity introduced before as

(34a) $$\mu =\frac{\mu_{\mathrm{eff}}}{\sqrt{1-4\upsilon^{2}}},$$

(34b) $$\eta_{s}=\frac{\mu_{\mathrm{eff}}}{2\omega \upsilon }.$$

**Figure 5:**
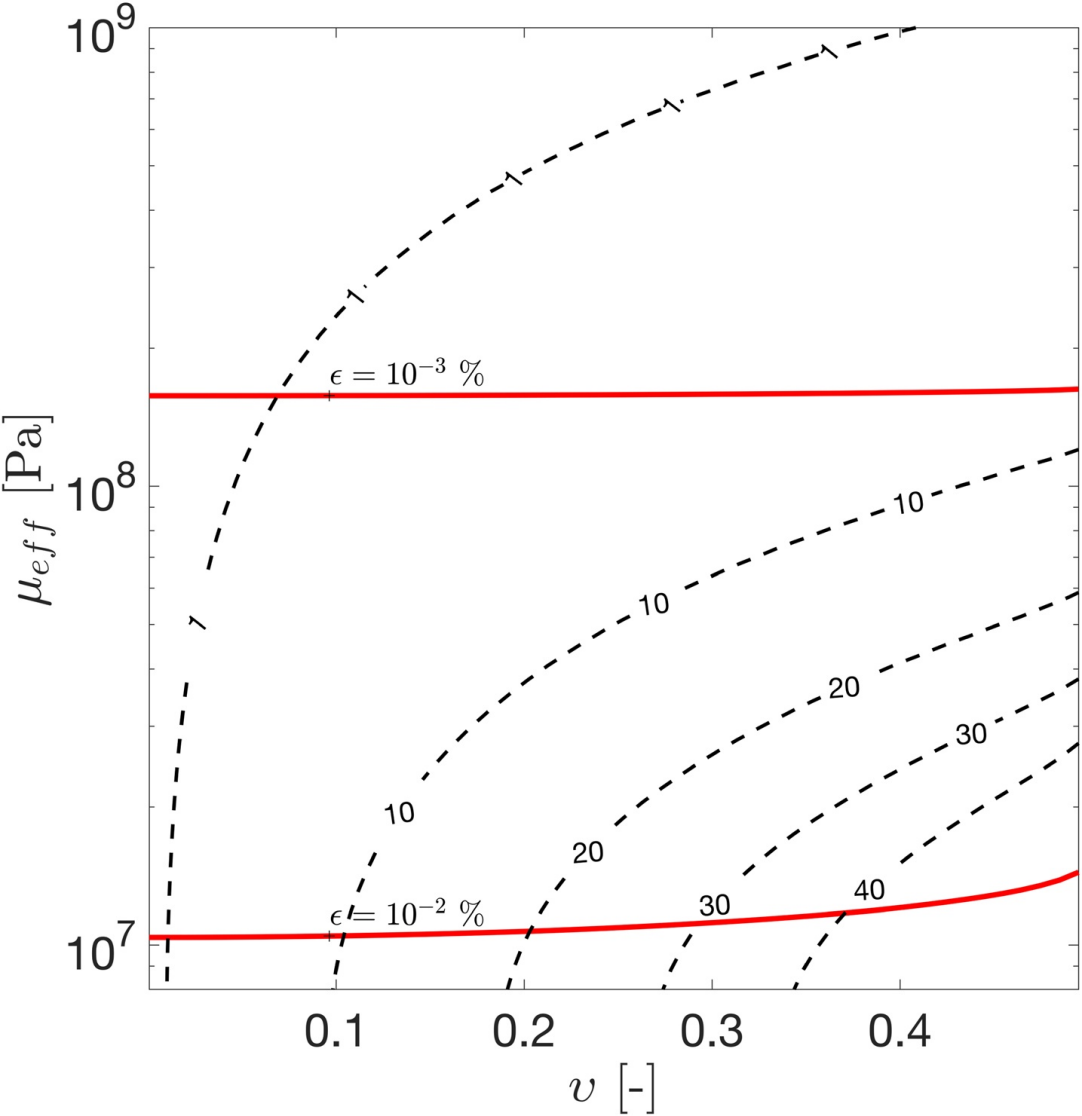
Total tidal dissipation in Enceladus' core for different values of core effective shear modulus and damping coefficient. A viscoelastic core and ice shell are assumed. The red lines indicate the maximum deviatoric strain attained within the core.

Figure 5 shows the amount of tidal dissipation in the core as function of *μ*_*eff*_ and *υ*. For *υ* > 0.1 more than 10 GW can be generated in Enceladus' core, provided the shear modulus is low enough. The values of the damping coefficient and effective shear modulus depend on factors that include the amplitude of the deviatoric strain, the confining pressure, and the forcing frequency (Faul & Jackson, 2005; Lambe & Whitman, 1969; Seed et al., 1986).

Goldreich and Sari (2009) showed that the effective rigidity of an unconsolidated body is smaller than that of a monolith due the concentration of stresses in sharp contact points. They proposed that the shear modulus is controlled by the curvature radius at these contact points, which in turn depends on the yield strain of the material *ϵ*_*Y*_, and showed that the effective rigidity of an unconsolidated body can be estimated as $\mu_{\mathrm{eff}}={\left(\frac{2g\rho R}{19}\frac{\mu }{\epsilon_{Y}}\right)}^{1/2}$, with *R* and *g* the surface radius and gravity and *ρ* the bulk density. This expression is consistent with an increase in shear modulus with confining pressure seen in the laboratory (Goddard, 1990). Using values representative of Enceladus's core (*μ* ∼ 1 GPa, *ϵ*_*Y*_ = 10^−2^), we find *μ*_*eff*_ ∼ 0.7 GPa—a value much higher than that required to attain high values of tidal dissipation for moderate values of damping coefficient *υ* (Figure 5).

Laboratory experiments can also be used to bound *υ* and *μ*_*eff*_. If the material experiences high deviatoric strains, *υ* increases. Laboratory data shows a pronounced increase of *υ* for *ϵ* > 0.01%; *υ* can reach values higher than 0.15 for *ϵ* > 0.1% (e.g., Seed et al., 1986; Rollins et al., 1998). For Enceladus' tidal amplitude, these high strains are only attained if the material has a low effective shear modulus (*μ*_*eff*_ ∼ 10^7^ − 10^8^ Pa) (Figure 5). The shear modulus increases with increasing overburden pressure and decreases with the amplitude of the deviatoric strain. For small strains (*ϵ* < 0.001%), the shear modulus of typical sand mixtures at Enceladus' core pressure (5–50 MPa) is on the order of 10^9^ Pa (Seed et al., 1986), incompatible with enhanced dissipation. Strains on the order of 0.01%–0.1% can reduce the effective shear modulus by around 50% (Rollins et al., 1998; Seed et al., 1986). Furthermore, the effective shear modulus is expected to decrease and the damping coefficient to increase at lower forcing frequencies (Faul & Jackson, 2005). Unfortunately, laboratory data is only available for a frequency range (0.01–1 Hz) much higher than Enceladus' tidal frequency (∼10^−5^ Hz). It remains to be seen if such changes are sufficient to access the high‐dissipation region of Figure 5. Laboratory experiments at Enceladus‐like conditions (high confining pressure, low forcing frequency) are required to assess whether Enceladus' core is in the highly deformable state required for the generation of the observed thermal activity.

A more direct measurement of Enceladus' core viscosity could be provided by a future Enceladan mission. Due to the viscosity of the core and the ice shell, their tidal responses are characterized by phase lags with respect to the forcing. The ice‐shell and core phase lags affect the gravity field and the surface displacements of the moon in different ways. Because the ocean decouples the ice shell from the core, the phase lag of the moon's surface displacement is mostly dependent on the viscosity of the ice shell; in contrast, the phase lag of the moon's gravity field depends on both the viscosity of the core and the ice shell. A low‐viscosity core leads to a large gravity phase lag but has a much smaller effect on the surface‐displacement phase lag; in contrast, a low viscosity ice shell produces phase lags in the gravity field and surface displacements of similar magnitude. Therefore, by measuring the difference between gravity and surface displacement phase lags, we can distinguish between a low‐viscosity and a high‐viscosity core. Hussmann et al. (2016) proposed this strategy to constrain Europa's core viscosity and the viscosity of high‐pressure ice layers within Ganymede; a similar technique could be used for Enceladus (Marusiak et al., 2021).

**Figure 6:**
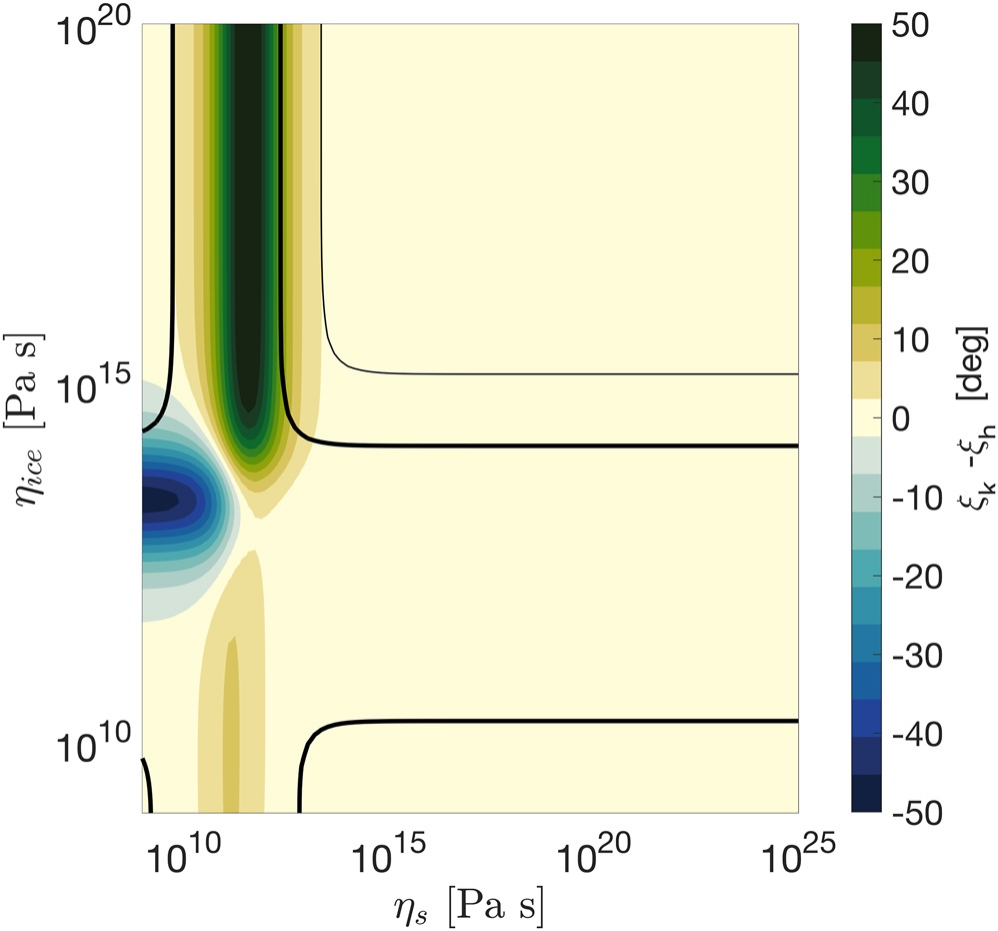
Difference in gravity and surface‐displacements phase lags for different values of core and ice shell viscosity. The thick and thin black contours show combinations of parameters for which tidal heating is 10 and 1 GW, respectively. A shear modulus of 1 GPa is assumed for the core; the properties of the ice shell are those given in Table 1, except for the viscosity, which we vary. Maxwell rheology is assumed for both ice shell and core.

The gravity and surface‐displacement phase lags are given by the phase lags of the gravitational and radial displacement Love numbers *k*_2_ and *h*_2_. Figure 6 shows the difference in gravity and surface‐displacement phase lags, $\xi_{k_{2}}-\xi_{h_{2}}$. If the core has a low viscosity, phase‐lag differences up to 50° are attained. This also holds if the more complex Andrade rheology is considered (Appendix F). The gravity phase lag $\xi_{k_{2}}$ could be measured by precise tracking of a single or dual orbiter around Enceladus (e.g., Ermakov et al., 2021). The surface‐displacements phase lag $\xi_{h_{2}}$ could be measured using a laser or radar altimeter (Steinbrügge et al., 2015, 2018). Such measurement would help to constrain Enceladus' core viscosity and settle the long‐standing puzzle of where Enceladus' heat is coming from.

## 4 Conclusions

Several observations suggest that Enceladus' core likely is a porous silicate matrix throughout which water can permeate. The tidal response of a porous core is expected to be markedly different from that of a non‐porous one: the presence of pores renders the body more compressible, pressure within the pores can alter the stress field, and water can flow within the core adding an additional dissipation mechanism. For a non‐porous core, tidal dissipation is only high if the core has a rigidity and a viscosity significantly lower than those expected for a monolithic silicate core. Recently, Liao et al. (2020) presented an analysis of the tidal response of Enceladus' porous core using Biot's theory of poroviscoelasticity and showed that poroviscoelastic effects can increase tidal dissipation for core properties compatible with those of silicates.

Liao et al. (2020) cautioned that their model relies in some assumptions that required further scrutiny: they considered the tidal response only of the core and ignored the overlying ocean and ice shell, neglected the effects of self‐gravity, and forced the problem via a prescribed surface strain rather than via the tidal potential. In this paper, we extended the model of Liao et al. (2020) by combining the theory of poroviscoelasticity (e.g., Biot, 1941; Cheng, 2016) with the theory commonly used to obtain the tidal deformation of viscoelastic, self‐gravitating bodies (e.g., Love, 1906; Sabadini et al., 2016).

With this theory, we assessed the relevance of the assumptions made by Liao et al. (2020), and studied the subspace of core properties for which tidal heating can explain Enceladus' thermal output. We started by considering a model of Enceladus' core without an overlying ocean and ice shell and showed that the boundary conditions at the core boundary play a central role. If the core is forced via a prescribed radial strain —as done in Liao et al. (2020)— tidal dissipation in both the solid and liquid phases can be orders of magnitude higher compared to that given by standard viscoelastic models. However, if a free surface is assumed (no‐stress) and the core is forced via a tidal body force, dissipation in both liquid and solid phases is reduced. In this scenario, Enceladus' thermal output can only be explained by tidal dissipation in the solid phase if the core viscosity is very low (*η*_*s*_ ∼ 10^9^–10^12^ Pa s) compared to that characteristic of silicates or via Darcian dissipation in the fluid if the core is highly permeable (*κ* > 10^−5^ m^2^). We then considered a more realistic multi‐layered model consisting of a porous core, a subsurface ocean and an ice‐shell. We showed that the presence of a hydrostatic ocean hinders the tidal response of the core. More importantly, tidal dissipation due to Darcy flow is severely reduced, making it difficult to reconcile tidal dissipation in Enceladus' rocky core with the observed thermal output.

Dissipation within the solid phase can still account for Enceladus' thermal output if the core is weak and has a low viscosity. This requirement appears to be incompatible with a monolithic, silicate core. Yet Choblet et al. (2017) ascribed the low shear modulus and viscosity to grain–grain friction in a fragmented core akin to a rubble pile. While it is true that a low viscosity can arise from this process, it only occurs if the tidal strain is sufficiently large, which requires a low shear modulus that is difficult to reconcile with laboratory experiments (Rollins et al., 1998; Seed et al., 1986). However, available laboratory data is not representative of Enceladus' core conditions, which points to the need for further laboratory work. Alternatively, we show that a future Enceladus mission could probe the core's viscosity by measuring the phase lag of tidally induced changes in the gravity field and surface deformation.

Other tidally active worlds might also have porous regions. Vigorous tidal heating can partially melt the mantle of a planet or a moon forming a porous sublayer filled with magma. Galileo's magnetometer data suggests that Io has a partially molten asthenosphere with a melt fraction of 20% (Khurana et al., 2011) (although alternative explanations have been proposed for the magnetometer data (Blöcker et al., 2018; Roth et al., 2017)). Io is the closest example of a magma‐rich world (Khurana et al., 2011; Peale et al., 1979; Spencer et al., 2020), but extrasolar worlds experiencing higher levels of tidal heating might also be common (e.g., Peters & Turner, 2013; Rovira‐Navarro et al., 2021). So far, attempts to compute the tidal response of bodies with a partially molten asthenosphere have either used the theory of viscoelasticity (e.g., Fischer & Spohn, 1990; Segatz et al., 1988), or the Laplace tidal equations commonly employed to model ocean tides (Hay et al., 2020; Tyler et al., 2015). Our model opens the door to study the tidal response of these worlds in a new light.

## Figures and Tables

**Figure F1:**
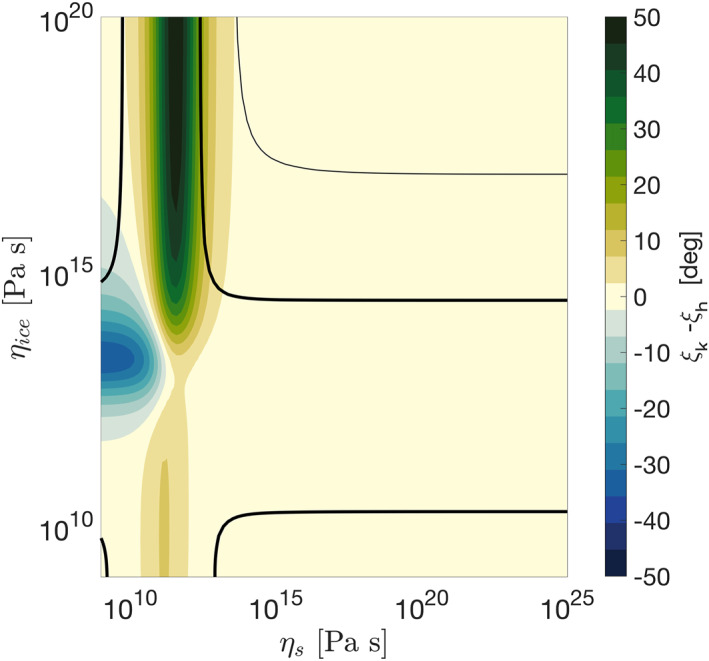
Same as Figure 6 but for Andrade rheology. *β* = 0.3 and *χ* = 1 are assumed for the ice shell and the core.

## Data Availability

The code developed for this manuscript and used to obtain the results presented here can be accessed in https://doi.org/10.5281/zenodo.6403046 (Rovira‐Navarro, 2022).

## Appendix A Solution Method

We solve Equations (29a), (29b), (30), (31), (32), (33) in the Fourier domain. Using the spherical symmetry of the problem, we decompose the Fourier‐transformed variables using spherical harmonics. Scalar fields such as ${\hat{\phi }}^{\Delta }$, ${\hat{\rho }}^{\Delta }$ are written using normalized real spherical harmonics $\left(Y_{l}^{m}\right)$ of degree *l* and order *m* as

(A1a)
$${\hat{\phi }}^{\Delta }\left(r,\theta ,\varphi \right)={\hat{\phi }}_{lm}^{\Delta }\left(r\right)Y_{l}^{m}\left(\theta ,\varphi \right),$$

(A1b)
$${\hat{\rho }}^{\Delta }\left(r,\theta ,\varphi \right)={\hat{\rho }}_{lm}^{\Delta }\left(r\right)Y_{l}^{m}\left(\theta ,\varphi \right),$$

with

(A2)
$$Y_{l}^{m}=\left\{\begin{array}{cc} {\left(-1\right)}^{m}\sqrt{2}\sqrt{\frac{2l+1}{4\pi }\frac{\left(l-|m|\right)!}{\left(l+|m|\right)!}}P_{l}^{\left|m\right|}\left(\cos\;\theta \right)\sin\left(|m|\varphi \right),\; & \text{if}\;m<0\\ \sqrt{\frac{2l+1}{4\pi }}P_{l}^{m}\left(\cos\;\theta \right),\; & \text{if}\;m=0\\ {\left(-1\right)}^{m}\sqrt{2}\sqrt{\frac{2l+1}{4\pi }\frac{\left(l-m\right)!}{\left(l+m\right)!}}P_{l}^{m}\left(\cos\;\theta \right)\cos\left(m\varphi \right),\; & \text{if}\;m>0 \end{array}\right.$$

$P_{l}^{m}$ are associated Legendre polynomials of degree *l* and order *m*.

The vector fields are similarly expanded using vector spherical harmonics:

(A3a)
$$\hat{u}\left(r,\theta ,\varphi \right)={\hat{u}}_{lm}\left(r\right)R_{l}^{m}+{\hat{v}}_{lm}\left(r\right)S_{l}^{m},$$

(A3b)
$$\hat{q}\left(r,\theta ,\varphi \right)={\hat{q}}_{lm}\left(r\right)R_{l}^{m}+{\hat{s}}_{lm}\left(r\right)S_{l}^{m}.$$

${\hat{u}}_{lm}$ and ${\hat{q}}_{lm}$, and ${\hat{v}}_{lm}$ and ${\hat{s}}_{lm}$ are the radial and tangential components of the displacement and flow field, respectively, and $R_{l}^{m}$ and $S_{l}^{m}$ are vector spherical harmonics,

(A4)
$$R_{l}^{m}=Y_{l}^{m}e_{r},\;S_{l}^{m}=\nabla_{\theta ,\varphi }Y_{l}^{m}.$$

Using the previous definitions and the constitutive equations, the governing equations can be cast into a first order differential equation of the form

(A5)
$$\frac{d\hat{y}}{dr}=A\hat{y}.$$

***A*** is a matrix given in Appendix B and $\hat{y}$ is a vector containing 8 radial functions:

(A6a)
$${\hat{y}}_{1}={\hat{u}}_{lm}$$

(A6b)
$${\hat{y}}_{2}={\hat{v}}_{lm}$$

(A6c)
$${\hat{y}}_{3}=\hat{\lambda }{\hat{\epsilon }}_{lm}^{V}-\alpha {\hat{p}}_{lm}^{\Delta }+2\hat{\mu }\frac{d{\hat{u}}_{lm}}{dr}$$

(A6d)
$${\hat{y}}_{4}=\hat{\mu }\left(\frac{d{\hat{v}}_{lm}}{dr}-\frac{{\hat{v}}_{lm}}{r}+\frac{{\hat{u}}_{lm}}{r}\right)$$

(A6e)
$${\hat{y}}_{5}={\hat{\phi }}_{lm}^{\Delta }$$

(A6f)
$${\hat{y}}_{6}=\frac{d{\hat{\phi }}_{lm}^{\Delta }}{dr}+\frac{l+1}{r}{\hat{\phi }}_{lm}^{\Delta }+4\pi G\rho_{0}{\hat{u}}_{lm}-\frac{4\pi G\rho_{l,0}\kappa }{i\omega \eta_{l}}\left(\frac{d}{dr}\left({\hat{p}}_{lm}^{\Delta }+\rho_{l,0}{\hat{\phi }}_{lm}^{\Delta }\right)+\frac{g\rho_{l,0}}{K_{l}}{\hat{p}}_{lm}^{\Delta }\right)$$

(A6g)
$${\hat{y}}_{7}={\hat{p}}_{lm}^{\Delta }$$

(A6h)
$${\hat{y}}_{8}=\frac{d}{dr}\left({\hat{p}}_{lm}^{\Delta }+\rho_{l,0}{\hat{\phi }}_{lm}^{\Delta }\right)+\frac{g\rho_{l,0}}{K_{l}}{\hat{p}}_{lm}^{\Delta }$$

${\hat{\epsilon }}_{lm}$ is the divergence of the displacement vector given by

(A7)
$${\hat{\epsilon }}_{lm}^{V}=\frac{d{\hat{y}}_{1}}{dr}+\frac{2}{r}{\hat{y}}_{1}-\frac{l\left(l+1\right)}{r}{\hat{y}}_{2}.$$

And $\hat{\mu }$ and $\hat{\lambda }$ are the complex rigidity and complex drained first Lamé parameter,

(A8a)
$$\hat{\mu }=\mu \frac{1}{1-\frac{i\mu }{\eta \omega }},$$

(A8b)
$$\hat{\lambda }=K-\frac{2}{3}\hat{\mu }.$$

Note that the radial functions ${\hat{y}}_{1-8}$ are the same radial functions commonly used to solve the viscoelastic tidal problem (e.g., Sabadini et al., 2016) except that two extra variables (${\hat{y}}_{7}$ and ${\hat{y}}_{8}$) are added, and ${\hat{y}}_{6}$ is modified. ${\hat{y}}_{1}$ and ${\hat{y}}_{2}$ are respectively the radial and tangential displacements, ${\hat{y}}_{3}$ and ${\hat{y}}_{4}$ correspond to the radial and tangential components of the stress tensor ($\sigma_{rr}^{\Delta }$, $\sigma_{\theta r}^{\Delta }$), ${\hat{y}}_{5}$ is the disturbing potential, and ${\hat{y}}_{6}$ is the so‐called potential stress. ${\hat{y}}_{7}$ is the pore pressure and ${\hat{y}}_{8}$ is proportional to the radial component of Darcy flow.

For incompressible internal liquid layers, it is sufficient to solve Laplace's equation. The problem can be similarly cast into a matrix form

(A9)
$$\frac{d\hat{z}}{dr}=A_{o}\hat{z}$$

where $z={\left({\hat{z}}_{5},{\hat{z}}_{6}\right)}^{T}$ and *z*_5_ and *z*_6_ are two radial functions corresponding to the perturbing potential and potential stress in the ocean:

(A10a)
$${\hat{z}}_{5}={\hat{\phi }}_{lm}^{\Delta },$$

(A10b)
$${\hat{z}}_{6}=\frac{d{\hat{\phi }}_{lm}^{\Delta }}{dr}+\left(\frac{l+1}{r}-\frac{4\pi G\rho_{0}}{g}\right){\hat{\phi }}_{lm}^{\Delta }.$$

***A***_*o*_ follows from Poisson's equation and is given in Appendix B.

The surface boundary conditions, Equations (23), (24), (25), (26), should be written in terms of the *y* functions. The stress boundary conditions at the surface are easily obtained. In contrast the boundary condition for the gradient of the tidal potential requires some further discussion. Using Equation 30 for the perturbing density, the Fourier‐transformed Equation 31 and the divergence theorem, Equation 24 can be written as

(A11)
$$\frac{\partial {\hat{\phi }}^{\Delta }\left(R+\delta \right)}{\partial r}-\frac{\partial {\hat{\phi }}^{\Delta }\left(R-\delta \right)}{\partial r}=4\pi \rho_{0}G\hat{u}\cdot e_{r}+4\pi G\frac{\rho_{l,0}}{\omega i}\hat{q}\cdot e_{r}.$$

We split the perturbing potential into the self‐gravity and the tidal potential components (*ϕ* ^*G*^, *ϕ* ^*T*^). In order to fulfill Poisson's equation outside of the body (*r* > *R*), *ϕ* ^*G*^ should be of the form

(A12)
$${\hat{\phi }}^{G}\left(r\right)=\phi^{G}\left(R\right){\left(\frac{r}{R}\right)}^{-l-1}.$$

Furthermore for a tidal forcing of degree *l* we have

(A13)
$${\hat{\phi }}^{T}\left(r\right)=\phi^{T}\left(R\right){\left(\frac{r}{R}\right)}^{l}.$$

Plugging Equations A12, A13 into Equation A11, expanding the different fields in spherical harmonics, using Darcy's law to compute ***q*** (Equation 29b), and taking the limit *δ* → 0, we get

(A14)
$${\hat{y}}_{6}\left(R\right)=\frac{2l+1}{R},$$

where we have assumed a unit tidal forcing, $\phi_{lm}^{T}\left(R\right)=1$. In a similar way, it can be shown that ${\hat{y}}_{6}$ is continuous at solid interfaces. The continuity of ${\hat{y}}_{6}$ and the fact that the boundary condition at the surface for the tidal forcing adopts a simple form, make using ${\hat{y}}_{6}$ convenient. Together with the stress boundary condition, the surface boundary conditions are

(A15a)
$${\hat{y}}_{3}\left(R\right)=0,$$

(A15b)
$${\hat{y}}_{4}\left(R\right)=0,$$

(A15c)
$${\hat{y}}_{6}\left(R\right)=\frac{2l+1}{R}.$$

As explained in Section 2.2 these boundary conditions are different than those used by Liao et al. (2020). Using our notation, Liao et al. (2020) surface boundary conditions translate to:

(A16a)
$$\frac{d{\hat{y}}_{1}\left(R\right)}{dr}={\hat{\epsilon }}_{rr,0},$$

(A16b)
$$\frac{d{\hat{y}}_{2}\left(R\right)}{dr}+\frac{{\hat{y}}_{2}\left(R\right)-{\hat{y}}_{1}\left(R\right)}{r}=0,$$

(A16c)
$${\hat{y}}_{6}\left(R\right)=0.$$

Equation  (12), (13a), (13b) imposes a prescribed strain of amplitude ${\hat{\epsilon }}_{rr,0}$, Equation (12), (13a), (13b) imposes an irrotational displacement field, and Equation  (12), (13a), (13b) sets the gravitational tidal perturbation to 0.

The boundary condition at the porous layer interfaces (Equations 25 and 26) are simply:

(A17a)
$${\hat{y}}_{8}=0,$$

(A17b)
$${\hat{y}}_{3}+{\hat{y}}_{7}=0.$$

Continuity of ${\hat{y}}_{1}-{\hat{y}}_{6}$ is assumed at the solid layers boundaries. In case an internal liquid layer is present, additional boundary conditions are introduced at solid‐liquid interfaces: the tangential stress vanishes, the radial stress is given by the difference between the radial displacement and an equipotential surface, and the disturbing potential is continuous but the potential stress is not (Greff‐Lefftz et al., 2000; Jara‐Orué & Vermeersen, 2011):

(A18a)
$${\hat{y}}_{4}\left(r\right)=0,$$

(A18b)
$${\hat{y}}_{1}\left(r\right)-\frac{{\hat{y}}_{3}\left(r\right)}{g\left(r\right)\rho_{\mathrm{ocean}}}+\frac{{\hat{y}}_{5}\left(r\right)}{g\left(r\right)}=0,$$

(A18c)
$${\hat{y}}_{5}\left(r\right)-{\hat{z}}_{5}\left(r\right)=0,$$

(A18d)
$${\hat{y}}_{6}\left(r\right)-{\hat{z}}_{6}\left(r\right)-4\pi G\rho_{\mathrm{ocean}}\left({\hat{y}}_{1}\left(r\right)+\frac{{\hat{z}}_{5}\left(r\right)}{g\left(r\right)}\right)=0.$$

Starting from the center of the moon or the liquid core‐mantle boundary (if the body in question has a liquid core), the previous equations are integrated radially using a Runge‐Kutta‐4 integrator (see Appendix C). Once the radial functions are obtained for the different components of the tidal potential, the displacement, flux, stress and strain fields can be computed as explained in Appendix D.

## Appendix B Propagation Matrix

The non‐zero elements of the propagation matrix ***A*** are:

(B1)
$$\begin{array}{cc} A_{11}=-2\frac{\hat{\lambda }}{\left(2\hat{\mu }+\hat{\lambda }\right)r} & A_{51}=-4\pi G\rho_{0}\\ A_{12}=l\left(l+1\right)\frac{\hat{\lambda }}{\left(2\hat{\mu }+\hat{\lambda }\right)r} & A_{55}=-\frac{l+1}{r}\\ A_{13}=\frac{1}{2\hat{\mu }+\hat{\lambda }} & A_{56}=1\\ A_{17}=\frac{\alpha }{2\hat{\hat{\mu }}+\hat{\lambda }} & A_{58}=\frac{4\pi G\rho_{l,0}\kappa }{i\omega \eta_{l}}\\ A_{21}=-\frac{1}{r} & A_{61}=-\frac{4\pi \left(l+1\right)G\rho_{0}}{r}\\ A_{22}=\frac{1}{r} & A_{62}=\frac{4\pi l\left(l+1\right)G\rho_{0}}{r}\\ A_{24}=\frac{1}{\hat{\mu }} & A_{65}=-\frac{4\pi l\left(l+1\right)G\rho_{l,0}^{2}\kappa }{i\omega \eta_{l}r^{2}}\\ A_{31}=\frac{4}{r}\left(\frac{\hat{\mu }}{r}\frac{3\hat{\lambda }+2\hat{\mu }}{2\hat{\mu }+\hat{\lambda }}-\rho_{0}g\right)+\frac{2g\alpha \rho_{l,0}}{r}\left(\frac{-\hat{\lambda }}{2\hat{\mu }+\hat{\lambda }}+1\right) & A_{66}=\frac{\left(l-1\right)}{r}\\ A_{32}=-\frac{l\left(l+1\right)}{r}\left(\frac{2\hat{\mu }}{r}\frac{2\hat{\mu }+3\hat{\lambda }}{2\hat{\mu }+\hat{\lambda }}-\rho_{0}g\right)+\frac{l\left(l+1\right)g\rho_{l,0}\alpha }{r}\left(\frac{\hat{\lambda }}{2\hat{\mu }+\hat{\lambda }}-1\right) & A_{67}=-\frac{4\pi l\left(l+1\right)G\rho_{l,0}\kappa }{i\omega \eta_{l}r^{2}}\\ A_{33}=\frac{1}{2\hat{\mu }+\hat{\lambda }}\left(-\frac{4\hat{\mu }}{r}+g\alpha \rho_{l,0}\right) & A_{68}=\frac{4\pi G\left(l+1\right)\rho_{l,0}\kappa }{i\omega \eta_{l}r}\\ A_{34}=\frac{l\left(l+1\right)}{r} & A_{71}=4\pi G\rho_{l,0}\rho_{0}\\ A_{35}=-\rho_{0}\frac{l+1}{r} & A_{75}=\rho_{l,0}\frac{\left(l+1\right)}{r}\\ A_{36}=\rho_{0} & A_{76}=-\rho_{l,0}\\ A_{37}=\frac{\alpha }{2\hat{\mu }+\hat{\lambda }}\left(-\frac{4\hat{\mu }}{r}+\rho_{l,0}g\alpha \right)+g\rho_{l,0}\left(\frac{\Phi }{K_{f}}+\frac{\alpha -\Phi }{K_{s}}\right) & A_{77}=-\frac{g\rho_{l,0}}{K_{l}}\\ A_{38}=\frac{4\pi G\rho_{l,0}\rho_{0}\kappa }{i\omega \eta_{f}} & A_{78}=1-\frac{4\pi G\rho_{l,0}^{2}\kappa }{i\omega \eta_{l}}\;\\ A_{41}=-\frac{1}{r}\left(\frac{\hat{\mu }}{r}\left(2+\frac{4\hat{\lambda }}{2\hat{\mu }+\hat{\lambda }}\right)-\rho_{0}g\right) & A_{81}=\frac{2i\alpha \omega \eta_{l}}{\kappa r}\left(1-\frac{\hat{\lambda }}{2\hat{\mu }+\hat{\lambda }}\right)\\ A_{42}=\frac{2\hat{\mu }}{r^{2}}\left(l^{2}+l-1+\frac{l\left(l+1\right)\hat{\lambda }}{\left(2\hat{\mu }+\hat{\lambda }\right)}\right) & A_{82}=-\frac{il\left(l+1\right)\alpha \omega \eta_{l}}{\kappa r}\left(1-\frac{\hat{\lambda }}{2\hat{\mu }+\hat{\lambda }}\right)\\ A_{43}=-\frac{1}{r}\left(1-\frac{2\hat{\mu }}{2\hat{\mu }+\hat{\lambda }}\right) & A_{83}=\frac{i\alpha \omega \eta_{l}}{\kappa \left(2\hat{\mu }+\hat{\lambda }\right)}\\ A_{44}=-\frac{3}{r} & A_{85}=\frac{l\left(l+1\right)\rho_{l,0}}{r^{2}}\\ A_{45}=\frac{\rho_{0}}{r} & A_{87}=\frac{l\left(l+1\right)}{r^{2}}+\frac{i\omega \eta_{l}}{\kappa }\left(\frac{\alpha^{2}}{2\hat{\mu }+\hat{\lambda }}+\frac{\Phi }{K_{f}}+\frac{\alpha -\Phi }{K_{s}}\right)\\ A_{47}=2\frac{\alpha \hat{\mu }}{r\left(2\hat{\mu }+\hat{\lambda }\right)} & A_{88}=\frac{-2}{r} \end{array}$$

The propagation matrix of a non‐porous incompressible material is recovered if *α* = 0, *Φ* = 0, *ρ*_*l*,0_ = 0 and $\hat{\lambda }\to \infty$. Also, by turning‐off self‐gravity (*ρ*_0_, *ρ*_*l*,0_ = 0) the equations used in (Liao et al., 2020) are recovered.

For the ocean propagator matrix ***A***_*o*_, we have (Saito, 1974):

(B2)
$$\begin{array}{cc} & A_{o,11}=\frac{4\pi G\rho_{0}}{g}-\frac{l+1}{r}\\ & A_{o,12}=1\\ & A_{o,21}=\frac{2\left(l-1\right)}{r}\frac{4\pi G\rho_{0}}{g}\\ & A_{o,22}=\frac{l-1}{r}-\frac{4\pi G\rho_{0}}{g} \end{array}$$

## Appendix C Propagating the Solution

We start integrating Equation A5 at the center of the body (*r*_0_ = 0), or at the core‐mantle interface (*r*_0_ = *r*_*c*_) if the moon has a liquid core. In either case, three integration constants $C_{0}={\left(C_{1},C_{2},C_{3}\right)}^{T}$ are introduced. The solution at *r*_0_ is $\hat{y}=B_{0}C_{0}$. ***B***_0_ is a matrix; if the body does not have a liquid core it is given by

(C1)
$$B_{0}=\left(\begin{array}{ccc} 0 & 0 & 0\\ 0 & 0 & 0\\ 1 & 0 & 0\\ 0 & 1 & 0\\ 0 & 0 & 0\\ 0 & 0 & 1\\ 0 & 0 & 0\\ 0 & 0 & 0 \end{array}\right),$$

otherwise, it is given by (Sabadini et al., 2016)

(C2)
$$B_{0}=\left(\begin{array}{ccc} -\frac{3r_{c}^{l-1}}{4\pi G\rho_{c}} & 0 & 1\\ 0 & 1 & 0\\ 0 & 0 & \frac{4\pi G\rho_{c}^{2}r_{c}}{3}\\ 0 & 0 & 0\\ r_{c}^{l} & 0 & 0\\ 2\left(l-1\right)r_{c}^{l-1} & 0 & 4\pi G\rho_{c}\\ 0 & 0 & 0\\ 0 & 0 & 0 \end{array}\right),$$

with *ρ*_*c*_ and *r*_*c*_ the core density and radius. An integration constant *C*_4_ is introduced for the porous layer so that *y*_7_(*r*_*p*_) = *C*_4_. At interfaces between solid layers continuity implies that

(C3)
$${\hat{y}}^{i}\left(r_{i}\right)=P{\hat{y}}^{i-1}\left(r_{i}\right)+\delta_{ip}B_{\mathrm{porous}}C_{4},$$

where *δ*_*ip*_ is the Kronecker delta, the superscript indicates the layer index –*p* being the index of the porous layer– and ***P*** and ***B***_*porous*_ are two matrices given by

(C4)
$$P=\left(\begin{array}{cccccccc} 1 & 0 & 0 & 0 & 0 & 0 & 0 & 0\\ 0 & 1 & 0 & 0 & 0 & 0 & 0 & 0\\ 0 & 0 & 1 & 0 & 0 & 0 & 0 & 0\\ 0 & 0 & 0 & 1 & 0 & 0 & 0 & 0\\ 0 & 0 & 0 & 0 & 1 & 0 & 0 & 0\\ 0 & 0 & 0 & 0 & 0 & 1 & 0 & 0\\ 0 & 0 & 0 & 0 & 0 & 0 & 0 & 0\\ 0 & 0 & 0 & 0 & 0 & 0 & 0 & 0 \end{array}\right),$$

and

(C5)
$$B_{\mathrm{porous}}={\left(0,0,0,0,0,0,1,0\right)}^{T}.$$

If an internal ocean is present, additional integration constants are introduced. At the ocean base, the solution is given by

(C6)
$$z\left(r_{o}\right)=IC_{\mathrm{ocean}}$$

with $C_{\mathrm{ocean}}={\left(C_{5},C_{6}\right)}^{T}$. At the upper‐boundary of the ocean (*r*_*o*_), the solution is

(C7)
$$y\left(r_{o}\right)=B_{\mathrm{ice}}C_{\mathrm{ice}}$$

with $C_{\mathrm{ice}}={\left(C_{7},C_{8},C_{9},C_{10}\right)}^{T}$ and ***B***_*ice*_ being

(C8)
$$B_{\mathrm{ice}}=\left(\begin{array}{cccc} 1 & 0 & -\frac{1}{g\left(r_{o}\right)} & 0\\ 0 & 1 & 0 & 0\\ \rho_{\mathrm{ocean}}g\left(r_{o}\right) & 0 & 0 & 0\\ 0 & 0 & 0 & 0\\ 0 & 0 & 1 & 0\\ 0 & 0 & 0 & 1\\ 0 & 0 & 0 & 0\\ 0 & 0 & 0 & 0 \end{array}\right).$$

Note that ***y*** (*r*_*o*_) already satisfies the boundary conditions (Equations A18a and A18b).

The integration constants *C*_1_ − *C*_10_ are obtained by propagating the solution using Equations A5 and A9 and applying the surface boundary conditions (Equations A15, A16 and (A17a), (A17b)), and, if the moon has an internal liquid layer, the boundary conditions at the solid‐liquid interfaces (Equation (A18a), (A18b), (A18c), (A18d)).

## Appendix D Building the Solution

Once the radial functions (***y***, ***z***) are obtained, they can be used to build the complete solution. The displacement and flow fields are:

(D1a)
$$\hat{u}\left(r,\theta ,\varphi \right)={\hat{y}}_{1}R_{l}^{m}+{\hat{y}}_{2}S_{l}^{m},$$

(D1b)
$$\hat{q}\left(r,\theta ,\varphi \right)=-\frac{\kappa }{\eta_{f}}{\hat{y}}_{8}R_{l}^{m}-\frac{\kappa }{\eta_{f}}\frac{1}{r}\left({\hat{y}}_{7}+\rho_{f}{\hat{y}}_{5}\right)S_{l}^{m}.$$

Similarly, the different components of the strain tensor are given by:

(D2a)
$${\hat{\epsilon }}_{rr}^{\Delta }\left(r,\theta ,\varphi \right)=\frac{d{\hat{y}}_{1}}{dr}Y_{l}^{m},$$

(D2b)
$${\hat{\epsilon }}_{\theta \theta }^{\Delta }\left(r,\theta ,\varphi \right)=\frac{1}{r}\left\{\left[{\hat{y}}_{1}-\frac{l\left(l+1\right)}{2}{\hat{y}}_{2}\right]Y_{l}^{m}+\frac{{\hat{y}}_{2}}{2}X_{l}^{m}\right\},$$

(D2c)
$${\hat{\epsilon }}_{\varphi \varphi }^{\Delta }\left(r,\theta ,\varphi \right)=\frac{1}{r}\left\{\left[{\hat{y}}_{1}-\frac{l\left(l+1\right)}{2}y_{2}\right]Y_{l}^{m}-\frac{{\hat{y}}_{2}}{2}X_{l}^{m}\right\},$$

(D2d)
$${\hat{\epsilon }}_{r\theta }^{\Delta }\left(r,\theta ,\varphi \right)=\frac{1}{2}\left[\frac{d{\hat{y}}_{2}}{dr}+\frac{{\hat{y}}_{1}-{\hat{y}}_{2}}{r}\right]\frac{\partial Y_{l}^{m}}{\partial \theta },$$

(D2e)
$${\hat{\epsilon }}_{r\varphi }^{\Delta }\left(r,\theta ,\varphi \right)=\frac{1}{2}\left[\frac{d{\hat{y}}_{2}}{dr}+\frac{{\hat{y}}_{1}-{\hat{y}}_{2}}{r}\right]\frac{1}{\sin\;\theta }\frac{\partial Y_{l}^{m}}{\partial \varphi },$$

(D2f)
$${\hat{\epsilon }}_{\theta ,\varphi }^{\Delta }\left(r,\theta ,\varphi \right)=\frac{{\hat{y}}_{2}}{2r}Z_{l}^{m},$$

with

(D3a)
$$X_{l}^{m}=2\frac{\partial^{2}Y_{l}^{m}}{{\partial \theta }^{2}}+l\left(l+1\right)Y_{l}^{m},$$

(D3b)
$$Z_{l}^{m}=2\frac{\partial }{\partial \theta }\left(\frac{1}{\sin\;\theta }\frac{\partial Y_{l}^{m}}{\partial \varphi }\right).$$

The different components of the stress tensor are:

(D4a)
$${\hat{\sigma }}_{rr}^{\Delta }=\hat{\lambda }{\hat{\epsilon }}_{lm}^{V}Y_{l}^{m}+2\hat{\mu }{\hat{\epsilon }}_{rr}^{\Delta }-\alpha {\hat{y}}_{7}Y_{l}^{m},$$

(D4b)
$${\hat{\sigma }}_{\theta \theta }^{\Delta }=\hat{\lambda }{\hat{\epsilon }}_{lm}^{V}Y_{l}^{m}+2\hat{\mu }\epsilon_{\theta \theta }^{\Delta }-\alpha {\hat{y}}_{7}Y_{l}^{m},$$

(D4c)
$${\hat{\sigma }}_{\varphi \varphi }^{\Delta }=\hat{\lambda }{\hat{\epsilon }}_{lm}^{V}Y_{l}^{m}+2\hat{\mu }{\hat{\epsilon }}_{\varphi \varphi }^{\Delta }-\alpha {\hat{y}}_{7}Y_{l}^{m},$$

(D4d)
$${\hat{\sigma }}_{r\theta }^{\Delta }=2\hat{\mu }{\hat{\epsilon }}_{r\theta }^{\Delta },$$

(D4e)
$${\hat{\sigma }}_{r\varphi }^{\Delta }=2\hat{\mu }{\hat{\epsilon }}_{r\varphi }^{\Delta },$$

(D4f)
$${\hat{\sigma }}_{\theta \varphi }^{\Delta }=2\hat{\mu }{\hat{\epsilon }}_{\theta \varphi }^{\Delta }.$$

The variation in fluid content *ζ* can be obtained using

(D5)
$$\hat{\zeta }=\alpha {\hat{\epsilon }}_{lm}^{V}Y_{l}^{m}+\frac{\alpha^{2}}{K_{u}-K_{d}}{\hat{p}}_{lm}^{\Delta }Y_{l}^{m}.$$

The approach presented above allows us to obtain the internal response of a moon to a unit tidal forcing of degree *l* and order *m* $\left(\phi^{T}=Y_{l}^{m}\right)$. For a satellite in an eccentric orbit, the tidal potential contains terms of degree 2 and orders 0, −2 and 2 (Equation 22). As the equations are linear, to obtain the tidal response we can compute the radial functions for *l* = 2 and combine them considering the amplitude of the different terms. To obtain the total tidal response for a given field $\hat{a}$ (e.g., displacement vector, stress tensor, strain tensor, etc.), we use

(D6)
$$\hat{a}\left(r,\theta ,\varphi \right)={\left(\omega R\right)}^{2}e\left(3\sqrt{\frac{\pi }{5}}{\hat{a}}_{20}\left(r,\theta ,\varphi \right)-3\sqrt{\frac{3\pi }{5}}{\hat{a}}_{22}\left(r,\theta ,\varphi \right)+4\sqrt{\frac{3\pi }{5}}i{\hat{a}}_{2-2}\left(r,\theta ,\varphi \right)\right),$$

where *a*_*lm*_ are the solution for degree *l* and order *m*. The solution in the time domain is

(D7)
$$a\left(r,\theta ,\varphi ,t\right)={\left(\omega R\right)}^{2}eRe\left\{\left(3\sqrt{\frac{\pi }{5}}{\hat{a}}_{20}\left(r,\theta ,\varphi \right)-3\sqrt{\frac{3\pi }{5}}{\hat{a}}_{22}\left(r,\theta ,\varphi \right)+4\sqrt{\frac{3\pi }{5}}i{\hat{a}}_{2-2}\left(r,\theta ,\varphi \right)\right)\exp\left(i\omega t\right)\right\}.$$

Once the solution is obtained, we can compute the volumetric energy dissipated in the solid,

(D8)
$${\dot{E}}_{v,solid}=-\frac{\omega }{2}\left[Re\left({\hat{\sigma }}^{\Delta }\right):Im\left({\hat{\epsilon }}^{\Delta }\right)-Im\left({\hat{\sigma }}^{\Delta }\right):Re\left({\hat{\epsilon }}^{\Delta }\right)+Re\left({\hat{p}}^{\Delta }\right)Im\left(\hat{\zeta }\right)-Im\left({\hat{p}}^{\Delta }\right)Re\left(\hat{\zeta }\right)\right],$$

and liquid phase,

(D9)
$${\dot{E}}_{v,liquid}=\frac{1}{2}\frac{\eta_{l}}{\kappa }\left(|{\hat{q}}_{r}{|}^{2}+|{\hat{q}}_{\theta }{|}^{2}+|{\hat{q}}_{\varphi }{|}^{2}\right).$$

The total energy dissipated in the solid and the liquid is obtained by numerically integrating Equations D8 and D9. Alternatively, it can also be found by plugging the radial functions $\left(\hat{y}\right)$ into (Equations (D1a), (D1b), (D2a), (D2b) and (D4a), (D4b), (D4c), (D4d), (D4e), (D4f)) and then using Equations D8 and D9. Using that

(D10)
$$\int_{S}R_{l}^{m}\cdot R_{l^{\prime }}^{m^{\prime }}dS=\delta_{l,l^{\prime }}\delta_{m,m^{\prime }}$$

and

(D11)
$$\int_{S}S_{l}^{m}\cdot S_{l^{\prime }}^{m^{\prime }}dS=l\left(l+1\right)\delta_{l,l^{\prime }}\delta_{m,m^{\prime }},$$

we obtain the angular averaged of the volumetric power $\left({\dot{E}}_{s}\right)$:

(D12a)
$${\dot{E}}_{s,solid}\left(r\right)=\frac{Im\left(\hat{\mu }\right)\omega }{8\pi r^{2}}\left(\frac{4}{3}|r\frac{d{\hat{y}}_{1}}{dr}-{\hat{y}}_{1}+l\left(l+1\right){\hat{y}}_{2}{|}^{2}\right.\left.+l\left(l+1\right){\left|\frac{r{\hat{y}}_{4}}{\hat{\mu }}\right|}^{2}+l\left(l+1\right)\left(l\left(l+1\right)-2\right)|{\hat{y}}_{2}{|}^{2}\right)$$

(D12b)
$${\dot{E}}_{s,liquid}\left(r\right)=\frac{1}{8\pi r^{2}}\frac{\kappa }{\eta_{l}}\left(r^{2}|{\hat{y}}_{8}{|}^{2}+l\left(l+1\right)|{\hat{y}}_{7}+\rho_{l,0}{\hat{y}}_{5}{|}^{2}\right)$$

The first expression is equivalent to that obtained by Beuthe (2013) and Tobie et al. (2005) for a viscoelastic body. The total tidal dissipation ${\dot{E}}_{t}$ can then be obtained by performing the radial integral of (Equation (D12a), (D12b)):

(D13)
$${\dot{E}}_{t}=4\pi \int_{0}^{R}r^{2}{\dot{E}}_{s}dr.$$

For a tidal forcing of the type given by Equation 22, we find

(D14a)
$${\dot{E}}_{t,solid}=\frac{336\pi^{2}}{5}\omega^{5}R^{4}e^{2}\int_{0}^{R}r^{2}{\dot{E}}_{s,solid}dr.$$

(D14b)
$${\dot{E}}_{t,liquid}=\frac{336\pi^{2}}{5}\frac{\omega^{4}R^{4}\kappa }{\eta_{l}}e^{2}\int_{0}^{R}r^{2}{\dot{E}}_{s,liquid}dr.$$

## Appendix E Incompressible Core Solution

If we consider that both the liquid and the porous matrix are incompressible (*K*_*s*_ → *∞*, *K*_*l*_ → *∞*), we can obtain an analytical expression for the flow field in the porous layer. Mass conservation implies that *ζ* → 0 (Equation 31), and the pore pressure field can simply be obtained by solving the Laplace equation (Equation (29a), (29b))

(E1)
$$\nabla^{2}\left(p^{\Delta }+\rho_{l,0}\phi^{\Delta }\right)=\nabla^{2}p^{*}=0.$$

with *p** a modified pressure. Solving Laplace's equation in spherical coordinates we find

(E2)
$$p^{*}=\left(A_{l}r^{l}+B_{l}r^{-l-1}\right)Y_{l}^{m}\left(\theta ,\varphi \right),$$

where $Y_{l}^{m}$ is a spherical harmonic of degree *l* and order *m* and *A*_*l*_ and *B*_*l*_ are two integration constants to be found using the boundary conditions. The solution should be regular at *r* = 0, which implies that *B*_*l*_ = 0. Additionally, at the boundary we impose the boundary conditions given by Equation 26 to find *A*_*l*_. If there is no ocean, we have *p**(*r*_1_) = *ρ*_*l*,0_ *ϕ* ^Δ^(*r*_1_); in contrast, if there is an ocean, we have *p**(*r*_1_) = *P*_*ocean*_(*r*_1_) + *ρ*_*l*,0_ *ϕ* ^Δ^(*r*_1_). The pressure at the core‐ocean boundary is given by the difference between the equipotential surface − *ϕ* ^Δ^/*g* and the radial displacement at the core ocean‐boundary *u*_*r*_(*r*_1_) (Equation (18a), (18b), (18c)). Using these boundary conditions, we can obtain *A*_*l*_ for a core with a free surface or one overlaid by an ocean:

(E3)
$$A_{l}^{\mathrm{free}}=\frac{\rho_{l,0}\phi_{l}^{\Delta }\left(r_{1}\right)}{r_{1}^{l}};\;A_{l}^{\mathrm{ocean}}=-h_{l}\left(r_{1}\right)\frac{\rho_{l,0}\phi_{l}^{\Delta }\left(r_{1}\right)}{r_{1}^{l}},$$

where *h*_*l*_ is the radial displacement Love number.

We can now use Equation D14b to compute the total amount of Darcian dissipation,

(E4a)
$${\dot{E}}_{\mathrm{liquid,t}}^{\mathrm{free}}=\frac{84}{5}\pi \frac{R_{c}^{5}\rho_{l,0}^{2}\omega^{4}\kappa e^{2}}{\eta_{l}},$$

(E4b)
$${\dot{E}}_{\mathrm{liquid,t}}^{\mathrm{ocean}}=\frac{84}{5}h_{2}^{2}\left(R_{c}\right)\pi \frac{R_{c}^{5}\rho_{l,0}^{2}\omega^{4}\kappa e^{2}}{\eta_{l}}.$$

The previous expressions provide a good approximation to the compressible cases presented in Section 3 (see Figure 2). Bearing in mind that |*h*_*l*_(*r*_1_)|≪ 1, we find that Darcy dissipation is severely reduced by the presence of an ocean.

## Appendix F Andrade Rheology

Alternative rheology laws can be introduced using the correspondence principle. If Andrade rheology is considered, the complex shear modulus (Equation (A18a), (A18b), (A18c), (A18d)), is given by

(F1)
$$\hat{\mu }={\left(\frac{1}{\mu }-\frac{i}{\eta \omega }+\frac{\mu^{\beta -1}\beta !}{{\left(i\eta \chi \omega \right)}^{\beta }}\right)}^{-1}.$$

*β* is a constant that takes values between 0.1 and 0.4 (e.g., Renaud & Henning, 2018) and *χ* a parameter that depends on the ratio between the anelastic and the Maxwell time (Efroimsky, 2012). If diffusion creep dominates, *χ* ≈ 1, which is commonly assumed for ices (Castillo‐Rogez et al., 2011; Rhoden & Walker, 2022; Shoji et al., 2013).

We obtain the gravity and surface‐displacement phase lags using the Andrade rheology (Equation F1), we use *β* = 0.3 and *χ* = 1 for both ice and rock. As in Shoji et al. (2013), we find that Andrade rheology increases tidal dissipation in the ice shell for viscosities with a Maxwell time higher than the forcing frequency. The difference in phase lag remains similar as those found using the Maxwell model (Section 3.3, Figure 6, Figure F1).
